# Stakeholder Perspectives on Humanistic Implementation of Computer Perception in Health Care: Qualitative Study

**DOI:** 10.2196/79182

**Published:** 2026-01-05

**Authors:** Kristin M Kostick-Quenet, Meghan E Hurley, Syed Ayaz, John D Herrington, Casey J Zampella, Julia Parish-Morris, Birkan Tunç, Gabriel Lázaro-Muñoz, Jennifer Blumenthal-Barby, Eric A Storch

**Affiliations:** 1 Center for Medical Ethics and Health Policy Baylor College of Medicine Houston, TX United States; 2 Department of Child and Adolescent Psychiatry and Behavioral Sciences Center for Autism Research Children's Hospital of Philadelphia Philadelphia, PA United States; 3 Department of Psychiatry Perelman School of Medicine University of Pennsylvania Philadelphia, PA United States; 4 Department of Neurosurgery Massachusetts General Hospital Boston, MA United States; 5 Menninger Department of Psychiatry and Behavioral Sciences Baylor College of Medicine Houston, TX United States

**Keywords:** computer perception, digital phenotyping, ethics, humanistic care, artificial intelligence, stakeholder engagement, context, consent, affective computing

## Abstract

**Background:**

Computer perception (CP) technologies—including digital phenotyping, affective computing, and related passive sensing approaches—offer unprecedented opportunities to personalize health care, especially mental health care, yet they also provoke concerns about privacy, bias, and the erosion of empathic, relationship-centered practice. At present, it remains elusive what stakeholders who design, deploy, and experience these tools in real-world settings perceive as the risks and benefits of CP technologies.

**Objective:**

This study aims to explore key stakeholder perspectives on the potential benefits, risks, and concerns associated with integrating CP technologies into patient care. A better understanding of these concerns is crucial for responding to and mitigating such concerns via design implementation strategies that augment, rather than compromise, patient-centered and humanistic care and associated outcomes.

**Methods:**

We conducted in-depth, semistructured interviews with 102 stakeholders involved at key points in CP’s development and implementation: adolescent patients (n=20) and their caregivers (n=20); frontline clinicians (n=20); technology developers (n=21); and ethics, legal, policy, or philosophy scholars (n=21). Interviews (~ 45 minutes each) explored perceived benefits, risks, and implementation challenges of CP in clinical care. Transcripts underwent thematic analysis by a multidisciplinary team; reliability was enhanced through double coding and consensus adjudication.

**Results:**

Stakeholders raised concerns across 7 themes: (1) Data Privacy and Protection (88/102, 86.3%); (2) Trustworthiness and Integrity of CP Technologies (72/102, 70.6%); (3) direct and indirect Patient Harms (65/102, 63.7%); (4) Utility and Implementation Challenges (60/102, 58.8%); (5) Patient-Specific Relevance (24/102, 23.5%); (6) Regulation and Governance (17/102, 16.7%); and (7) Philosophical Critiques of reductionism (13/102, 12.7%). A cross-cutting insight was the primacy of context and subjective meaning in determining whether CP outputs are clinically valid and actionable. Participants warned that without attention to these factors, algorithms risk misclassification and dehumanization of care.

**Conclusions:**

To operationalize humanistic safeguards, we propose “personalized road maps”: co-designed plans that predetermine which metrics will be monitored, how and when feedback is shared, thresholds for clinical action, and procedures for reconciling discrepancies between algorithmic inferences and lived experience. Road maps embed patient education, dynamic consent, and tailored feedback, thereby aligning CP deployment with patient autonomy, therapeutic alliance, and ethical transparency. This multistakeholder study provides the first comprehensive, evidence-based account of relational, technical, and governance challenges raised by CP tools in clinical care. By translating these insights into personalized road maps, we offer a practical framework for developers, clinicians, and policy makers seeking to harness continuous behavioral data while preserving the humanistic core of care.

## Introduction

### Computer Perception Tools in Mental Health Care

Computer perception (CP) tools, including digital phenotyping, affective computing, computational behavioral analysis, and other approaches that entail continuous and passive data collection using wearables and smartphone sensing, have been positioned as a remedy for longstanding diagnostic and informational gaps in mental health care. The term “computer perception” references the artificial intelligence (AI) subfield of computer “vision” but acknowledges a wider range of perceptive modalities beyond vision alone (eg, “hearing” through microphones, motion detection through accelerometers), referring not only to sensory acquisition but also to a system’s capacity to interpret, classify, and act upon such data—analogous to human perceptual processing that integrates recognition and interpretation. By leveraging sensors already embedded in everyday devices, these systems promise scalable, accessible surveillance of behaviors, as well as mood, cognition, and sociability, potentially addressing medicine’s chronic reliance on infrequent patient self-reports and clinician observation to gain insights into psychosocial, behavioral, and physiological states [1,2]. Although this study centers on mental health care, the ethical and translational issues examined here (ie, around inference, interpretation, and the integration of perceptual data into care) extend to other domains of medicine where continuous data streams are increasingly used for diagnosis and decision-making. CP tools also promise a personalized and patient-tailored diagnostic and therapeutic approach, in line with precision medicine goals [3-5]. Early studies suggest that CP-derived markers can forecast relapse in bipolar disorder, detect prodromal psychosis, tailor just-in-time behavioral prompts, and potentially widen access to mental health care. Yet the very features that make CP appealing also expose patients to unprecedented privacy risks, algorithmic bias, and a potential erosion of empathic, relationship-centered care [1,6-8].

Ethicists, regulators, and frontline stakeholders caution that integrating such pervasive sensing into care can imperil core values of confidentiality, fairness, and relational trust [9-12]. These impacts can be exacerbated by opaque algorithms, unclear pathways for secondary data reuse, and difficulties in obtaining meaningful informed consent in continuous monitoring scenarios. A limited number of studies [13-15] provide a foundation for understanding some of these concerns; however, no empirical research to date offers a comprehensive view of the wide-ranging perspectives held by diverse stakeholder groups regarding the benefits and risks of integrating CP into care. This study addresses this gap through an empirical exploration of diverse stakeholder perspectives, with special attention to impacts on humanistic, relationship-centered care.

The rationale for focusing on humanistic care is to underscore that good care, whether technological or manual, depends on recognizing the patient as a person with values, context, and dignity. Humanistic and humanized care frameworks [16-18] remind us that respectful dialogue, cultural sensitivity, and patient partnership are interwoven into the moral fabric of good practice [19]. Whether CP ultimately augments or erodes that fabric depends on how well designers, clinicians, and regulators anticipate the spectrum of ethical concerns voiced by those who will build, deploy, or live with these systems. This study, therefore, turns to those diverse stakeholders—developers, clinicians, patients, caregivers, and ethics, legal, and policy scholars—to ask how their concerns can guide the integration of CP in ways that preserve, rather than diminish, the humanization of care. While mental health provides a particularly vivid setting for exploring these questions, the concerns articulated by participants resonate across many areas of health care and health technology innovation.

### Background

What makes CP technologies unique is that they increasingly involve algorithmic *inferences* about a person’s moment-to-moment mental or sociobehavioral state, or about predicted outcomes such as mood relapse, suicidality, or treatment response [2,12,20,21]. These inferences are enabled by the ingestion of vast amounts of behavioral, physiological, and environmental signals from (usually) ordinary connected devices such as smartphones and wearables. Less often, they may involve implantable systems that continuously monitor physiological [22,23] or neural activity [24]. In psychiatric contexts, the approach is often called *digital phenotyping,* entailing the use of smartphones, wearables, and ambient sensors to stream accelerometry, GPS traces, keystroke dynamics, speech acoustics, heart rate variability, and other passively captured metadata. Those streams are preprocessed and feature-engineered [25,26] and then fed into statistical or deep learning models. Parallel work in *affective computing* [27] extends the approach to facial microexpressions, vocal prosody, or text sentiment to classify discrete emotions or arousal levels in real time [28].

As CP systems sit at the intersection of pervasive sensing and advancements in AI, they raise many of the same ethical issues highlighted in broader AI systems. Concerns about algorithmic bias, transparency, explainability, interpretability, fairness, and other aspects of “trustworthy” AI [29,30] are relevant. The rarity with which CP tools are validated on large, diverse validation cohorts means that algorithmic performance is likely to vary dramatically across demographic and clinical groups, raising reliability concerns and potentially amplifying health disparities [31]. Critics have also warned against overreliance on algorithmic inferences about patients’ health status [32,33], especially in “black box” systems that resist clinical scrutiny and accountability and compromise informed clinical decision-making [34]. Others [15,35] underscore legal uncertainties surrounding liability in cases of error, patient harm, or mismanagement of outputs or other feedback. The US National Institute of Standards and Technology’s AI Risk Management Framework [36] and the European Union AI Act [37] both categorize health-related CP tools as “high risk,” demanding rigorous safety, fairness, and oversight provisions.

Similar to other AI systems, CP tools thrive on voluminous datasets, not only across individuals but also for each individual, often referred to as individual “big data” or “deep data” [38]. Ethical critiques thus consistently foreground privacy vulnerabilities associated with sensitive behavioral data [13,14,39,40], and there is expert consensus [13] around the need for privacy and innovative consent approaches. Scholars (eg, [35,41] and C Deeney, BA et al, unpublished data, August 2024) caution against unwanted or involuntary disclosure to third parties, such as insurers or employers, especially in scenarios where data are controlled by consumer-grade device companies. Dynamic consent models have also been proposed [40] to replace onetime or broad consent approaches with ongoing, granular permissions; however, feasibility remains challenging [42].

### Challenges for Humanistic Care

Critics [9,43,44] have also converged on a deeper worry: as algorithms assume a larger share of the responsibility to observe and listen, the relational core of care risks being reduced to a “metrics management” exercise, where clinicians and patients spend their limited time consulting data trends rather than discussing the patient’s lived experience and therapeutic goals. Clinicians fear that multimodal dashboards could displace narrative dialogue, shifting the burden of self-monitoring and, by extension, responsibility for changes in functioning onto patients in ways that compromise dignity and mutual trust [9,45-47] and overprioritize technological over humanistic solutions [48]. Some warn that automated detection and treatment of illness may weaken the rapport and goal alignment that bolster the therapeutic alliance, unless paired with explicit, empathic communication strategies [49].

A limited set of empirical work reinforces these cautions. One study [47] documented mental health clinician enthusiasm for gaining rich, real-time insights but also highlighted concerns about workflow overload and the potential for automation bias, that is, deferring to algorithmic outputs even when they conflict with a clinician’s intuitions or a patient’s lived story. Another study [46] highlighted clinicians’ concerns that prioritizing passive data trends over self-reported narratives or active responses to clinical assessments could reduce opportunities for patients to reflect on their mental health, leading to diminished patient engagement. Experts [9,21] have raised flags that such asymmetries can tilt encounters toward dehumanization and require careful planning and implementation to achieve the goal of making otherwise invisible patterns visible and clinically useful.

These relational stakes bring long-standing ethical principles into focus and urge clinicians and researchers to keep dignity, empathy, patient empowerment, and shared decision-making at the forefront of clinical care. However, it remains unclear how best to do this in ways that engage multiple and often competing perspectives. Our study addresses this gap by exploring the range of concerns through interviews with over 100 stakeholders who design, deploy, and are the intended users of CP technologies. We catalogue considerations that extend beyond well-elaborated privacy and bias debates to the less operationalized relational harms that data-centric care may impose. By situating these concerns within established humanistic frameworks of dignity, empathy, and shared decision-making [17,18], we offer an anticipatory road map for researchers, developers, clinicians, and patients. The goal is not merely to identify technical fixes, but to ensure that as CP systems mature, they deepen rather than diminish the person-centered relationships that remain the centerpiece of care.

## Methods

### Study Design

As part of a 4-year study funded by the National Center for Advancing Translational Sciences (R01TR004243), we conducted in-depth, semistructured interviews (total n=102), including adolescent patients (n=20) and caregiver (n=20) dyads, clinicians (n=20), developers (n=21), and ethics, legal, policy, and philosophy scholars (n=21), to explore their perspectives on potential benefits, risks, and concerns around the integration of CP technologies into care.

### Participants

Respondents were recruited from a “sister” study (5R01MH125958) aiming to validate CP tools designed to quantify objective digital biobehavioral markers of socioemotional functioning. Participants included a clinical sample of adolescents (aged 12-17 years) with varied diagnoses, including autism, Tourette, anxiety, obsessive-compulsive disorder, and attention-deficit/hyperactivity disorder, as well as their caregivers (typically biological parents; Table 1). Diagnostic presentations for all adolescents were confirmed by expert providers using established clinical measures. Adolescent-caregiver dyads were referred to the study by the sister study’s coordinator and then contacted by a research assistant via phone or email to schedule an interview. Clinicians and developers (Table 2) were identified through an online literature search and existing professional networks. Participants were interviewed between January 2023 and August 2023.

**Table 1:** Demographics for interviewed adolescents and caregivers.^a^

Demographics			Adolescents (n=20), n (%)		Caregivers (n=20), n (%)		Total (N=40), n (%)		
**Gender**									
	Male	12 (60)		2 (10)		14 (35)			
	Female	8 (40)		18 (90)		26 (65)			
**Race**									
	American Indian or Alaska Native	0 (0)		1 (5)		1 (3)			
	Asian	1 (5)		1 (5)		2 (5)			
	Native Hawaiian or Other Pacific Islander	0 (0)		0 (0)		0 (0)			
	African American/Black	5 (25)		4 (20)		9 (23)			
	White	17 (85)		15 (75)		32 (80)			
**Ethnicity**									
	Hispanic or Latino	4 (20)		2 (10)		6 (15)			
	Not Hispanic or Latino	16 (80)		18 (90)		34 (85)			
**Marital status**									
	Married and living with spouse	N/A^b^		13 (65)		13 (33)			
	Widowed	N/A		1 (5)		1 (3)			
	Divorced	N/A		4 (20)		4 (10)			
	Separated	N/A		1 (5)		1 (3)			
	Never Married	N/A		1 (5)		1 (3)			
**Education level**									
	High school only or less	N/A		0 (0)		0 (0)			
	Trade school/associate’s degree	N/A		2 (10)		2 (5)			
	Bachelor’s degree	N/A		10 (50)		10 (25)			
	Master’s degree	N/A		4 (20)		4 (10)			
	Doctoral degree	N/A		4 (20)		4 (10)			
**Parental status**									
	Biological parent	N/A		18 (90)		18 (45)			
	Step parent	N/A		0 (0)		0 (0)			
	Adoptive parent	N/A		2 (10)		2 (5)			
**Diagnosed condition**									
	Obsessive-compulsive disorder	4 (20)		N/A		4 (10)			
	Autism	5 (25)		N/A		5 (13)			
	Attention-deficit/hyperactivity disorder	3 (15)		N/A		3 (8)			
	Anxiety	4^c^ (20)		N/A		4 (10)			
	Tourette	1 (5)		N/A		1 (3)			
	No clinical diagnosis or symptoms	9 (45)		N/A		9 (23)			
Average age, mean (SD)			14.9 (2.2)		48.3 (6.4)		N/A		

^a^Values may not total 100% owing to overlapping categories (eg, comorbidities), nonmutually exclusive response options, and skipped questions.

^b^N/A: not applicable.

^c^1 self-reported.

**Table 2:** Demographics for interviewed clinicians, developers, and ELPP.^a^

Demographics		Clinicians (n=20), n (%)	Developers (n=21), n (%)	Scholars (n=21), n (%)	Total (N=62), n (%)
**Gender**					
	Male	10 (50)	18 (86)	16 (76)	44 (55)
	Female	10 (50)	3 (14)	5 (24)	18 (29)
**Race**					
	American Indian or Alaska Native	0 (0)	0 (0)	0 (0)	0 (0)
	Asian	4 (20)	1 (5)	2 (10)	7 (11)
	Native Hawaiian, Pacific Islander, or Other	0 (0)	0 (0)	0 (0)	0 (0)
	African American/Black	0 (0)	1 (5)	0 (0)	1 (2)
	White	14 (70)	12 (57)	16 (76)	42 (68)
	Unreported/unknown	3 (15)	6 (29)	4 (19)	13 (21)
**Ethnicity**					
	Hispanic or Latino	0 (0)	0 (0)	1 (5)	1 (2)
	Not Hispanic or Latino	17 (85)	13 (62)	16 (76)	46 (74)
	Unreported/unknown	3 (15)	7 (33)	4 (19)	14 (23)
**Profession**					
	Clinician	3 (15)	N/A^b^	N/A	3 (5)
	Clinician-researcher	14 (70)	N/A	N/A	14 (23)
	Clinician-developer	3 (15)	4 (19)	N/A	7 (11)
	Developer	N/A	17 (81)	N/A	17 (34)
	Ethicist	N/A	N/A	6 (29)	6 (10)
	Lawyer	N/A	N/A	4 (19)	4 (6)
	Philosopher	N/A	N/A	1 (5)	1 (2)
	Other	N/A	N/A	10 (48)	10 (16)
**Specialty**					
	Psychiatry	7 (35)	N/A	N/A	7 (11)
	Psychology	7 (35)	N/A	N/A	7 (11)
	Neuroscience	4 (20)	N/A	N/A	4 (6)
	Industry	N/A	15 (71)	N/A	15 (24)
	Academic	N/A	3 (14)	N/A	3 (5)
	Cross-Sector	N/A	3 (14)	N/A	3 (5)
	Ethics	N/A	N/A	6 (29)	6 (10)
	Law	N/A	N/A	4 (19)	4 (6)
	Philosophy	N/A	N/A	1 (5)	1 (2)
	Other	2 (10)	N/A	10 (48)	12 (16)

^a^Some values may not total the number of stakeholders per group or 100% because certain responses were missing, some response options were nonmutually exclusive, and respondents were allowed to skip questions.

^b^N/A: not applicable.

### Data Collection

Separate but parallel interview guides were developed for all stakeholders, with the same constructs explored across groups, including perceived benefits and concerns regarding integrating CP tools into clinical care; impacts on care; attitudes toward automatic and passive detection of emotional and behavioral states; perceived accuracy and potential for misinterpretation, misattribution, or misclassification of symptoms or conditions; clinical utility and actionability; data security and privacy concerns; potential for unintended uses; perceived generalizability and potential for bias; and other emergent concerns. These domains were chosen based on issues raised in the clinical and ethics literature and with guidance from experienced bioethicists and mental health experts. Initial drafts of the interview guides were piloted with 2 psychologists (EAS and CJZ) specializing in adolescent mental health, resulting in minor clarifications in wording. Interviews were conducted via a secure videoconferencing platform (Zoom for Healthcare; Zoom Communications, Inc) and lasted an average of ~45 minutes. Participants watched a brief 1.5-minute “explainer” video defining CP as denoting AI systems (devices + algorithms) that not only sense but also infer and act upon multimodal behavioral and physiological signals. Demographic items were included to explore possible sociodemographic variation in perspectives and to facilitate downstream analyses or meta-analytic comparisons. Participants could select more than 1 racial or ethnic category, and no participant was required to respond to any demographic question.

### Ethics Approval

This study was reviewed and approved by the Baylor College of Medicine Institutional Review Board (H-52227), which waived the requirement for written consent, as the research procedures (interviews, deidentification of transcripts, and storage on secure servers) involved minimal risk to participating stakeholders; thus, participants provided verbal consent. Minors provided assent with parental consent. Identifiable participant information was stored behind a university firewall in a password-protected system with 2-factor authentication. All results are reported in aggregate and not linked to any identifiable participants, including in supplementary documents. All participants also completed a brief demographic questionnaire in REDCap (Research Electronic Data Capture; Vanderbilt University) via an emailed link.

### Data Analysis

Interviews were audio-recorded, transcribed verbatim, and analyzed using MAXQDA software (VERBI Software). Led by a qualitative methods expert (KMKQ), team members developed a codebook to identify thematic patterns in adolescent and caregiver responses to the topics described above. Each interview was coded by merging the work of 2 separate coders to reduce interpretability bias and enhance reliability. All team members received extensive training in qualitative analysis before participating in coding. We used thematic content analysis [50,51] to inductively identify themes by progressively abstracting relevant quotes, a process that entails reading every quotation to which a given code was attributed, paraphrasing each quotation (primary abstraction), further identifying which constructs were addressed by each quotation (secondary abstraction), and organizing constructs into themes. The multidisciplinary team responsible for thematic analysis consisted of the principal investigator (KMKQ), who is a medical anthropologist and bioethicist with expertise in qualitative and mixed methods research, bioethics, and the social and ethical dimensions of AI and digital phenotyping; and 3 research associates—2 master’s-level researchers with backgrounds in psychology, neuroscience, bioethics, and cognitive science, and 1 postbaccalaureate researcher with training in psychology and computer science. This combination of disciplinary and methodological perspectives was intentionally designed to reduce interpretive homogeneity and promote reflexivity. To enhance the validity of our findings, all abstractions were validated by at least one other member of the research team. In rare cases where abstractions reflected different interpretations, members of the research team met to reach consensus. Coding meetings emphasized interpretive dialogue rather than consensus by conformity, ensuring that thematic reliability reflected triangulation across diverse epistemic standpoints rather than agreement among individuals with similar expectations. Frequencies were also calculated for each theme by counting the number of individuals within each stakeholder group who contributed at least one quote coded under that theme. These frequencies and percentages are presented solely as descriptive indicators and are not intended to imply statistical significance or support inferential claims.

## Results

### Themes Identified

Stakeholders raised a wide range of concerns around the following themes (Table 3): (1) Trustworthiness and Integrity of CP Technologies (72/102, 70.6%; Multimedia Appendix 1); 2) Patient-Specific Relevance (24/102, 23.5%; Multimedia Appendix 2); (3) Utility and Implementation Challenges (60/102, 58.8%; Multimedia Appendix 3); (4) Regulation and Governance (17/102, 16.7%; Multimedia Appendix 4; (5) Data Privacy and Protection (88/102, 86.3%; Multimedia Appendix 5); (6) Patient Harms (65/102, 63.7%; Multimedia Appendix 6); and (7) Philosophical Critiques (13/102, 12.7%; Multimedia Appendix 7). All themes and subthemes are elaborated below, with illustrative quotations in the associated Multimedia Appendices 1-7.

**Table 3:** Theme frequencies.^a^

Theme	Developers (n=21), n (%)	Clinicians (n=20), n (%)	Adolescents (n=20), n (%)	Caregivers (n=20), n (%)	Ethics, law, policy, and philosophy scholars (n=21), n (%)	Total (N=102), n (%)
Trustworthiness and Integrity	17 (81)	15 (75)	8 (40)	15 (75)	17 (81)	72 (70.6)
Patient-Specific Relevance	3 (14)	3 (15)	3 (15)	7 (35)	8 (38)	24 (23.5)
Utility and Implementation	15 (71)	15 (75)	4 (20)	9 (45)	17 (81)	60 (58.8)
Regulation and Governance	4 (19)	4 (20)	0 (0)	1 (5)	8 (38)	17 (16.7)
Data Privacy and Protection	16 (76)	17 (85)	16 (80)	20 (100)	19 (90)	88 (86.3)
Patient Harms	9 (43)	16 (80)	4 (20)	18 (90)	18 (86)	65 (63.7)
Philosophical Critiques	2 (10)	2 (10)	0 (0)	2 (10)	7 (33)	13 (12.7)

^a^Frequencies and percentages are calculated within groups except for when they are in the “Total” column, where they are calculated across groups.

### Trustworthiness and Integrity of CP Technologies

#### Data Quality Constraints and Confounds

Developers, more than other stakeholder groups, raised concerns about the reliability of data streams from consumer-grade devices, emphasizing that variations in user behavior and differences in hardware performance can make it difficult to distinguish true physiological changes from sensor-related errors. They cautioned that without standardized protocols for device calibration and data collection, models built on such inputs may fail when deployed across different environments or patient populations.

#### Algorithmic Bias and Generalizability

Participants across all stakeholder groups also raised concerns about additional forms of algorithmic bias. Several scholars noted that many AI models are trained on relatively homogenous datasets, limiting their generalizability to more diverse populations. As these datasets often disproportionately represent individuals from more privileged groups (eg, younger, healthier, or majority-ethnic cohorts), algorithms may underperform or misclassify signals in marginalized communities. Participants further cautioned that unequal access to digital health technologies can skew training data even more, reinforcing systemic biases and potentially excluding the very populations most likely to benefit from improved care.

#### Construct Validity

Clinicians, developers, and scholars alike cautioned that the diagnostic constructs and clinical assessment tools used to validate most CP tools often lack strong links to clinically meaningful phenomena and fail to accommodate transdiagnostic symptom presentations, cultural and contextual variability, and temporal fluctuations in mental health. As a result, the digital markers derived from these tools risk remaining insufficiently grounded. Participants emphasized the need for rigorous validation studies to ensure that digital biomarkers accurately reflect patient states and that any interventions based on these measures are anchored in well-established clinical evidence.

### Patient-Specific Relevance

#### Accounting for Heterogeneity in Symptom Expression and Subjectivity

Stakeholders consistently emphasized that any use of digital health tools must first account for the immense diversity in how individuals experience and express their health and then situate those signals within each person’s unique context. Respondents across groups cautioned that a one-size-fits-all algorithm may miss or misinterpret patients who exhibit emotional or behavioral states differently from others; for example, some noted that while certain individuals express distress outwardly, others internalize such feelings, rendering them “invisible” to CP tools searching for external markers. Others added that accurate interpretation often depends on integrating multiple data streams; heart rate alone, for instance, may not distinguish stress from exercise without information about the broader context or behavioral pattern.

#### Accounting for Context and Meaning

Patients and caregivers, more than other groups, raised concerns that algorithms cannot effectively account for the rich social and cultural factors that shape patients’ experiences and behaviors, or how patients assign meaning to their symptoms and events. Some also emphasized the importance of proximate contextual features, such as fluctuations tied to work demands, family stressors, or lifestyle changes. Patients, in particular, worried that algorithms might draw conclusions based on fleeting or temporary signals rather than longer-term trends. Respondents across groups cautioned that such “decontextualized” metrics lack the construct validity required for clinical actionability, as they are likely to reflect inferences stripped of subjective meaning and, therefore, clinical significance.

### Utility and Implementation Challenges

#### Role of CP in Clinical Care

Stakeholders from all groups voiced a set of interrelated concerns about how CP tools are integrated into clinical workflows. Scholars and clinicians cautioned that clinicians may lean too heavily on algorithmic outputs, risking a form of “deskilling” in which they stop rigorously scrutinizing the data for quality or epistemic inconsistencies. They warned that clinicians may begin to accept CP suggestions uncritically (automation bias), thereby sidelining the human, relational interpretations developed through patient-provider dialogue.

#### Managing Risk and Liability

Clinicians, more than other groups, highlighted the dual dangers of missed events and overalerting. They noted that false negatives—instances where the system fails to detect deterioration—could leave patients unprotected, while excessive false positives could overwhelm clinicians and erode confidence in the tool, ultimately undermining patient safety rather than enhancing it. Clinicians also raised concerns about whether they may eventually be expected to use CP tools as these systems continue to evolve, or held liable if they choose not to, thereby compromising their autonomy in clinical decision-making.

#### Barriers to Utility

All stakeholder groups stressed that CP outputs must be interpretable and meaningful in real-world contexts to be actionable. Clinicians emphasized that data trends and inferences should be delivered through intuitive summaries and visualizations, accompanied by concise, actionable recommendations. They noted that this is complicated by the fact that the clinical significance of data trends may vary from one situation to another (see the “Patient-Specific Relevance” section), making consistent interpretation challenging. Developers and clinicians also raised concerns about the potential for confirmation bias, in which users may selectively interpret or emphasize data that confirm their expectations, thereby undermining the goal of these technologies to contribute novel informational value to clinical assessments.

### Regulation and Governance

#### Unclear or Insufficient Regulatory Frameworks

Clinicians and scholars, more than other groups, described 2 distinct but related regulatory challenges. First, many CP applications can (and in their view, should) fall under existing clinical-use regulations, such as those governing medical devices; yet, few concrete guidelines exist for implementing these requirements in practice. Ethics and policy experts noted that when CP tools nominally qualify as regulated devices, organizations may feel more comfortable adopting them; however, the absence of clear, step-by-step governance pathways often leaves developers and clinicians uncertain about how to operationalize data privacy, security, and ethical review processes. Second, participants emphasized that a large swath of CP technologies occupies a “regulatory gray zone” due to their overlap with devices in the consumer “wellness” sector, particularly those that collect passive or contextual data outside traditional care encounters. Scholars worried that without specifying oversight requirements for continuous, ambient monitoring, regulators risk leaving patients exposed to unvetted algorithms and unclear lines of accountability.

#### Responsibility for Ethical Technology Development and Compliance

Developers, scholars, and clinicians primarily expressed concerns about how innovation pressures interact with ethical safeguards. On the one hand, experts described the burden of balancing innovation against regulatory demands, noting that small teams sometimes struggle to absorb the time and cost required for formal ethics and security reviews. They also raised concerns about the deployment of closed-source, proprietary algorithms, which are often faster to market but opaque. These were contrasted with open-source alternatives, which permit external audit but come with greater technical support obligations. Across both choices, questions about liability were raised, with respondents arguing that without explicit legal clarity, neither developers nor health care providers know with certainty who would be held accountable if CP assessments lead to harm.

#### Need for Stakeholder Involvement

Respondents from all groups expressed strong consensus that regulation and governance structures must be co-designed with the people intended to benefit from these technologies. Ethics scholars argued that embedding patients’ and caregivers’ lived experiences into standards setting is vital to ensuring that tools address real-world needs. Clinicians highlighted the importance of rigorously interrogating when and under what circumstances CP outputs truly matter to patient care, rather than assuming that technological assessments will always be relevant. Participants across groups also called for interdisciplinary collaboration among technologists, clinicians, ethicists, and end users to bridge gaps in expertise, surface hidden risks, and develop governance models that are both practical and ethically robust.

### Data Privacy and Protection

#### Consent and Awareness

Patients described anxiety about unwanted or unintended disclosure of intimate behavioral and physiological data, noting that continuous collection can feel like a privacy breach. Other stakeholder types likewise questioned the appropriateness of capturing real-time location or mental health indicators, characterizing such practices as invasive and, in some cases, “creepy.” This unease was compounded by awareness that elements of coercion may come into play: individuals could feel pressured to share their data so as not to jeopardize access to health care services. Adding to these worries, stakeholders noted that explanations of data practices are often obscure, leaving patients unaware or uncertain about what exactly they are consenting to, who may access their data, what inferences could be drawn from it, and what kinds of feedback to expect. As a result, patients may be ill-equipped to make informed decisions about engaging with these CP tools or about what types of feedback to receive or decline (eg, exercising a “right not to know”).

Many participants, especially researchers, clinicians, and ethics scholars, criticized current informed consent practices as outdated and one-dimensional. They noted that patients typically encounter a single form at the outset of care (broad consent) without fully understanding the breadth of data being collected or the myriad ways it might later be used. Several respondents urged a shift toward dynamic consent models, in which patients receive clear, ongoing explanations and can granularly and dynamically opt in or out of specific data uses. They emphasized that such processes—which treat consent as an evolving conversation—are better suited to the continuous, ecological monitoring characteristic of CP approaches.

#### Secondary Use and Misuses

Many patients and caregivers reported being comfortable with primary clinical uses of CP data but expressed concern about secondary applications and potential misuses. Stakeholders across groups noted that, without clear legal protections, patient information could be repurposed for discriminatory profiling or accessed by commercial actors, with existing regulations offering little guidance on how to manage these downstream uses. They argued that the commodification and monetization of personal behavioral and physiological data, in the absence of robust data protection frameworks, could erode patient and caregiver trust in clinicians and health care systems and discourage future participation in digital health programs.

#### Monitoring and Surveillance

Stakeholders also observed that when individuals feel monitored rather than supported, they may withhold information, worry about data misuse, and question their providers’ trustworthiness. This concern may be particularly relevant for vulnerable populations, such as people experiencing psychosis, who may perceive passive monitoring as surveillance, and older adults who may have difficulty using wearables and apps—highlighting the need for adaptive protocols, additional safeguards, and alternative engagement strategies that respect each patient’s autonomy and comfort. They emphasized that passive monitoring can shift the experience from feeling supported to feeling observed, an effect that may be especially pronounced among vulnerable groups; for example, people experiencing psychosis may interpret continuous tracking as intrusive surveillance, and members of historically exploited populations may hold significant reservations.

### Patient Impacts and Harms

#### Overview

Stakeholders highlighted numerous ways in which the above concerns may translate into direct or indirect harms for patients:

#### Harms Due to Inaccurate or Premature Diagnoses

Stakeholders from all groups cautioned that algorithmic assessments delivered without sufficient clinical context can trigger a cascade of inappropriate interventions. They warned that acting on false positives or early “flags” could expose patients to unnecessary tests, treatments, or stigma long before a human expert has had an opportunity to validate the finding. They also noted the potential negative impacts when algorithmic conclusions diverge from patients’ own perceptions and experiences, creating conflict without clear pathways for resolution.

#### Diminished Human Connection in Health Care

A recurring theme, particularly among clinicians and patients, was the potential breakdown of the human connection in health care. Many stakeholders noted that an overreliance on data-driven CP tools could transform care into a more transactional and less empathetic process. Clinicians especially underscored the importance of maintaining therapeutic relationships grounded in respect, empathy, and alliance, warning that digital tools—while potentially efficient—could diminish the “human touch” that is central to healing. Many patients and caregivers echoed this concern, fearing that health care interactions could become increasingly impersonal. Scholars and clinicians also discussed the potential for digital health tools to contribute to epistemic injustice, whereby patients’ lived experiences may be undervalued in comparison to data-driven assessments. Some stakeholders expressed concern that an emphasis on objective data could lead clinicians to discount patients’ subjective experiences—especially in complex domains such as mental health, where self-reports already face considerable scrutiny. They warned that such dismissal could erode patient autonomy and contribute to a dehumanization of care, particularly if clinicians and patients allow algorithmic inferences to assume an increasingly prominent role relative to human judgment in decision-making.

#### Responsibility Shifts and “Empowerment” Pitfalls

Another significant concern raised by clinicians involved the shifting of responsibility from health care providers to patients. As digital tools increasingly monitor and manage health, patients are often expected to assume a larger role in their own care. While some viewed this shift as empowering, many clinicians feared it could overwhelm patients—especially those without the skills, knowledge, or interest to interpret continuous data feedback—potentially leading to confusion, stress, and unintended burdens.

Ethics scholars also noted that although the rhetoric of “empowerment” is often used to promote these tools, it can effectively shift responsibility onto individuals—particularly those with greater resources—while leaving vulnerable populations with few mechanisms to address complex health inequalities. They emphasized that this shift not only places an undue burden on patients to manage their health independently but also predisposes them to blame when improvements do not occur, potentially worsening feelings of shame or anxiety. Several ethics and policy scholars argued that this trend is reinforced by the technology sector’s tendency to view patients as consumers rather than individuals needing care, thereby framing health management as an individual rather than a collective responsibility.

Additionally, clinicians noted the risk that patients may defer responsibility to technology—such as smartphones—under the assumption that these tools will manage their health for them, which can diminish active engagement in their own care. They cautioned that when patients come to believe that their devices will “speak” on their behalf, they may become less inclined, and over time less able, to reflect on and articulate their own experiences and behavioral patterns.

#### Access Inequities and Disproportionate Burdens to Vulnerable Populations

Clinicians and scholars voiced further concerns about the potential of CP tools to exacerbate inequities and disproportionately burden vulnerable populations. Scholars emphasized that marginalized groups—including those experiencing poverty, homelessness, and other forms of marginalization—may be excluded from the benefits of these technologies due to a lack of access or capacity. For example, individuals without consistent access to, or familiarity with, technology might struggle to effectively use or trust these tools, limiting potential benefits and skewing training datasets in ways that perpetuate harmful biases and further exacerbate inequities.

Further, caregivers and ethicists, in particular, raised significant reservations about CP tools being leveraged or co-opted for surveillance, especially in communities with a history of being monitored, such as psychiatric and other vulnerable groups. Pressured consent emerged as another concern, particularly for individuals in lower social positions who might feel compelled to use these tools despite discomfort or uncertainty. Finally, stakeholders highlighted the risk of involuntary monitoring or detention, noting that misdiagnoses or inaccurate data could lead to wrongful decisions with severe consequences for individuals’ rights and treatment.

#### Threats to Privacy and Self-Determination

Stakeholders from all groups voiced strong concerns about the threats to privacy and autonomy posed by digital health tools. They highlighted the potential misuse of sensitive health data and the lack of transparency in how such information is collected and used. Scholars emphasized the need for stronger regulatory frameworks to ensure that patients’ privacy is protected and that they retain control over their personal health data. They warned that without adequate safeguards, the widespread adoption of these technologies could lead to breaches of trust and unauthorized access to sensitive information.

Clinicians noted that certain patient populations are likely to be disproportionately affected by these concerns and may require particularly robust clinical justifications, as well as enhanced protections or alternative approaches, to ensure that CP tools benefit their care while safeguarding their rights to self-determination and protection against discrimination.

#### Epistemic Injustice and Deprioritization of Patient Voices

Stakeholders cautioned that CP tools risk sidelining patients’ own experiences by privileging algorithmic inferences over first-person testimony. Ethics scholars noted that even highly accurate systems can produce outputs that contradict a patient’s self-knowledge, potentially leading clinicians to discount lived perceptions and destabilize trust. Caregivers emphasized that real-time observations—such as a parent’s instinct about a child’s well-being—must carry equal or greater weight than sensor data to avoid silencing those closest to the patient.

#### Overemphasis on Self-Optimization

Experts warned that voluntary self-tracking can evolve into a cultural expectation, similar to how smartphones have become indispensable. What begins as clinically guided monitoring risks morphing into relentless personal optimization, pressuring individuals to engage in continuous self-surveillance. Stakeholders argued that blurring the line between medical indication and consumer-driven tracking reduces complex human experiences to mere data points and undermines broader notions of well-being that cannot be quantified.

### Philosophical Critiques of CP

#### CP Is Insufficient to Capture Emotional States

Certain scholars cautioned that CP technologies cannot fully capture the rich complexity of human emotion. They argued that feelings are not reducible to physiological impulses or static signals, but instead unfold in nuanced, dynamic patterns that resist algorithmic measurement.

#### CP Cannot Infer Emotion via Behavior

Relatedly, some stakeholders emphasized that CP tools cannot reliably infer emotion from behavior alone. While sensors can record facial movements, voice acoustics, heart rate fluctuations, and other behavioral or physiological signals, these outward markers do not necessarily reflect internal experience and always require human interpretation. One scholar likened this need for interpretation to how a radiologist must analyze and contextualize an image.

#### CP Algorithms Embed Human Biases

Other participants emphasized that, because CP algorithms inevitably incorporate human biases, they cannot serve as purely objective indicators of pathology. They noted that every algorithm is trained on manually labeled data and thus carries forward the cultural assumptions and biases of its creators. They argued that reliance on precoded categories can obscure these underlying prejudices by presenting CP outputs as seemingly “objective.”

#### CP Inferences Are Not More Valuable Than Subjective Patient Insights

Some scholars challenged the overprioritization of data over dialogue, emphasizing that personal narratives—rooted in lived, phenomenological experience—provide primary and indispensable insights into illness that digital metrics cannot replace. They contended that patient testimony must “stand on equal footing” with any algorithmic outputs.

#### CP Reflects Techno-Solutionism

Scholars warned that addressing illness primarily through a technological lens reflects a broader misconception that technology can solve all problems. They emphasized the importance of attending to the social, political, and cultural dimensions of health. These stakeholders argued that an overemphasis on what can be measured or automated risks shaping health care interventions around the capabilities of machines rather than the holistic needs of people.

## Discussion

### Corroborating Existing Recommendations

Our investigation highlights the broad and varied concerns of diverse stakeholders—developers, clinicians, patients, caregivers, and ethics and policy experts—regarding the integration of CP into clinical care. Understanding and addressing these concerns is critical for designing implementation strategies that enhance, rather than compromise, patient-centered and humanistic care. Many of the themes echo longstanding critiques of data-centrism in medicine: CP represents the latest iteration of placing ever richer “deep data” streams at the center of care, now amplified by powerful AI and machine learning analytics. Accordingly, stakeholders reiterate familiar principles from the trustworthy AI framework, including explainability, interpretability, bias mitigation, fairness, and transparency. The opaque, “black-box” nature of many proprietary CP algorithms further compounds these challenges, leaving patients and caregivers without clear evidence of how inferences about mood, cognition, or behavior are generated. Respondents in our study, echoing prior calls, advocate for robust, domain-specific validation standards, enhanced algorithmic transparency, liability frameworks for errors, mechanisms for contesting outputs, and guidance on reliably interpreting CP results across diverse clinical settings. These imperatives are neither new nor contested; there is a broad consensus on the need for trustworthy algorithms coupled with humanistic care.

Similarly, the call for implementation frameworks that protect clinician judgment, patient agency, and the therapeutic alliance is well established. Stakeholders cautioned that uncritical, algorithm-driven monitoring risks displacing empathic dialogue by prioritizing decontextualized or biased metrics over patients’ own narratives, shifting the therapeutic focus from shared understanding to automated inference. These concerns are most pronounced for CP systems that directly infer diagnosis (classification) or prognosis (prediction), but may be less significant when CP is used to surface raw patterns—such as sleep or activity metrics—for human-guided interpretation. For example, rather than allowing an algorithm to label sleep patterns as pathological, clinicians could use a patient’s baseline sleep data—compared with population benchmarks—to ask, “What’s keeping you up at night?” and collaboratively determine what constitutes normal sleep for that individual in the context of work, family, or lifestyle factors. D’Alfonso and colleagues [9] describe this distinction as “manual” versus “AI-driven” use of CP, emphasizing the degree of human involvement in interpreting data. At the time of writing, most CP tools are not yet robust enough to rely solely on AI-driven inferences and therefore require substantial human interpretation to be clinically useful. However, as we argue elsewhere [52], this may not always remain the case; following the trajectory of AI in other domains, CP algorithms are likely to evolve to provide valid, accurate, patient-specific, and trustworthy inferences. Establishing humanistic approaches well in advance is a widely recognized and consensus goal.

### Novel Insights: The Importance of Context and Subjectivity

Our respondents highlighted 2 fundamental considerations for effectively and humanely integrating CP tools into care that have not been fully addressed elsewhere: the importance of context and subjectivity in determining the clinical significance of CP outputs. Stakeholders across all groups emphasized that observable behaviors—such as steps, voice tone, and facial micro-movements—are clinically actionable only when clinicians understand what those behaviors signify for the individual producing them and how the surrounding context shapes that meaning.

This caution echoes the “Theory of Constructed Emotion” proposed by Barrett et al [53] and supported by like-minded scholars [54-57], who challenge the classical view that emotions are biologically hard-wired states expressed through universal behavioral markers. Instead, the brain constructs each feeling from past experiences, cultural learning, and moment-to-moment interpretation; the same smile, for example, can signify joy, embarrassment, or compliance depending on context [28,58]. When CP systems infer affect solely from facial features, vocal prosody, heart rate variability, or other external cues, they risk reducing this complexity to generic labels—an error that disproportionately misinterprets individuals across different cultures, age groups, or clinical presentations [59].

To counter such reductionism, future CP strategies must integrate subjective meaning and environmental context alongside sensor data. Technically, this involves pairing passive streams with structured self-report or ecological annotations that capture the patient’s interpretation of events and the situational factors influencing them. Operationally, it requires structured conversations—from the earliest visits through follow-up—that identify which symptoms most constrain a person’s quality of life and how those symptoms might be detected digitally. The “Digital Measures That Matter to Patients” framework proposed by Manta and colleagues [60] provides concrete guidance, linking *meaningful aspects of health* to sensor-derived concepts of interest, outcomes, and end points within a patient-centered hierarchy.

In practice, applying this framework could mean, for example, that a patient who values uninterrupted sleep over daytime mood stability prioritizes actigraphy-based sleep metrics, whereas another concerned about social withdrawal might ask the system to flag sustained reductions in communication patterns. By integrating patient narratives and contextual details into metric selection and interpretation, clinicians can transform CP from a one-size-fits-all detector into a context-aware, individually tailored decision-support tool—remaining faithful to the subjective richness that stakeholders emphasize must never be lost.

### A Prototype for Humanistic Care With CP

#### Personalized Road Maps for CP Integration

To address these challenges, we introduce the concept of personalized road maps [61] for integrating CP into clinical care—a structured, co-designed plan that embeds humanistic values at every stage of digital phenotyping. Rather than treating data feedback as a series of discrete disclosures, personalized road maps are collaboratively developed by patients, caregivers, and clinician-researchers at the point of consent. Together, they specify the following:

- *Which metrics* (eg, activity patterns, speech markers, sleep variability) will be monitored and shared
- *When and how* these data will be returned—whether in real time, during clinic visits, through periodic summaries, or some strategic (nonarbitrary) mix of approaches
- *Thresholds for action,* delineating what combinations of signals should trigger outreach, referral, or adjustment of treatment
- *Conflict resolution procedures* for managing epistemic conflicts when CP outputs diverge from a patient’s self-report or a clinician’s judgment.

This iterative framework balances patient agency with clinical and ethical guardrails, inviting patients to contribute lived knowledge (eg, recognizing that reduced SMS text messaging often precedes mood dips), while researchers share their clinical expertise. Together, both parties anticipate and develop shared understandings of how their perspectives may be enriched by predictive insights from CP data trends and inferences. This approach reflects a view, articulated by others [49], that technology and humane care are not mutually exclusive, but can, in fact, be symbiotic. The personalized road map is designed to foster that symbiosis, serving as a living decision-support tool that aligns computational power with at least three operationalized, person-centered goals of care, including those listed below.

#### Empowerment and Shared Decision-Making

By inviting patients to coselect which CP signals matter most and how they wish to receive feedback, personalized road maps transform passive monitoring into an active partnership. This builds on Schmidt and D’Alfonso’s [47] finding that clinicians and clients value systems where patients can “switch off” sensors, control data sharing, and iteratively refine monitoring parameters. Patients can collaboratively choreograph the timing, dose, and content of feedback to align with their treatment goals. Embedding these choices upstream helps prevent downstream surprises or distress when digital inferences arise.

#### Trust and Therapeutic Alliance

Clear, cocrafted expectations—about what data will be returned, when, and under what conditions—help mitigate nocebo effects and overreliance on opaque risk scores. As Nghiem et al [46] observed, passive patient-generated health data are most useful when presented at clinically meaningful moments, rather than overwhelming clinicians in real time. Personalized road maps can specify this timing, ensuring that data review occurs within empathetic, dialogic encounters rather than disrupting them.

#### Ethical Transparency and Anticipation of Conflict

Documenting both the inclusion and exclusion of specific CP metrics is inspired by the “open notes” movement, providing patients with insight into the analytic process. This approach preserves their right to understand which factors shape their treatment pathways, as well as their right “not to know” certain inferences that might be counterproductive to clinical progress. Road maps also embed anticipatory strategies for epistemic conflicts. For example, if a wearable flags elevated stress while a patient reports feeling calm, the road map can offer coidentified strategies to guide the clinician and patient through a respectful dialogue about potential device errors, contextual factors, or unrecognized symptoms, rather than defaulting to algorithmic authority or privileging patient report.

### Innovating Consent for CP Approaches

As CP technologies transition from clinical research into routine care, these road maps will support clinical teams in their fiduciary responsibility to educate patients about anticipated benefits and risks, while transparently conveying areas of uncertainty. Enhancing existing consent procedures should begin with identifying the knowledge needs of patients and caregivers to enable truly informed consent. In a recent publication, we reported the results of an empirical, qualitative analysis [62] exploring the perspectives of adolescent patients and their caregivers participating in clinical laboratory research involving extensive CP data collection. Our findings demonstrated that patients and caregivers have information needs spanning 7 key themes: (1) clinical utility and value; (2) evidence, explainability, evaluation, and contestation; (3) accuracy and trustworthiness; (4) data security, privacy, and potential misuse; (5) patient consent, control, and autonomy; (6) the physician-patient relationship; and (7) patient safety, well-being, and dignity. A separate analysis (C Deeney, BA et al, unpublished data, August 2024) found that most patients and caregivers consider CP data highly sensitive and are reluctant to share these data beyond their clinical teams. While many participants expressed trust in existing data protections to safeguard CP data, they often misunderstood or overestimated the extent to which protections such as the Health Insurance Portability and Accountability Act (HIPAA) apply. Based on these findings, we proposed 5 key strategies: (1) educating patients on the limitations of existing data protections; (2) conducting targeted research, including forensic analyses, into secondary data exchanges to identify privacy breaches or reidentification risks; (3) enacting regulations that mandate greater transparency in health data transactions; (4) implementing computational mechanisms, such as distributed ledger technologies, to enhance data traceability and auditability; and (5) adopting dynamic consent models that allow patients to continuously manage and update their consent preferences.

Other scholars have similarly argued that static, onetime signatures are inadequate for the continuous, highly contextual data streams generated by CP tools. A systematic review of ethical considerations for passive data sensing [63] proposed interactive informed consent interfaces that allow participants to add social annotations, “talkback” questions, and multimodal visual aids—features shown to enhance comprehension and engagement [64,65]. Others have called for context-sensitive consent models [66,67], allowing patients to recalibrate permissions as circumstances change and enabling built-in data expiration options, so individuals can set automatic sunset dates [68]. These consent innovations should be embedded within the personalized road map architecture to ensure that consent remains an evolving, rather than static, agreement.

### Operationalizing Humanistic Use of CP

Most would agree that maintaining a sense of humanity in care is critical—and in fact, we already have a reasonably clear vision of what humanistic practice entails, even if current systems fall short. Humanistic care is compassionate, respectful, and empathetic. It is also collaborative, culturally sensitive, and empowering. The formative research presented here corroborates a substantial body of prior work [69-71] demonstrating how diverse stakeholders conceptualize and idealize humanistic care. In other words, further studies to delineate what constitutes humanistic practice and to demonstrate its benefits for patients, clinicians, and communities are no longer the priority; that foundational work has already been done. What is now required is rigorous, context-specific evidence identifying which CP integration strategies most effectively embody these established humanistic care ideals—that is, which organizational policies, device design features, relational practices, and value-based attitudes to incorporate, and which to eschew. We still lack evidence‑based guidelines for integrating CP, and the only way to develop them is to investigate a wide spectrum of implementation contexts to determine which combinations of features produce desired outcomes, for which patients, and under what circumstances. Our analysis highlights several feature domains that require systematic evaluation:

- *Data handling:* collection methods, governance structures, and privacy safeguards
- *Feedback logistics:* cadence, routing, and escalation pathways
- *Patient support:* education, engagement, and shared‑decision tools
- *Analytics:* modeling choices, interpretive aids, and decision‑support mechanisms
- usability, accessibility, and visualization elements
- *Workflow integration:* infrastructure requirements and task allocation
- *Clinician readiness:* training, supervision, and capacity building

Each domain contains multiple variables whose effects may differ by setting. Treating these variables as elements of a “constellation” and iteratively testing how their configurations influence clinical and humanistic outcomes will allow us to identify the scenarios in which specific approaches add value—and those in which they do not. Such empirical investigation may reveal that CP approaches are not suitable for every patient or clinical scenario.

### Concluding Reflections

Integrating CP technologies into everyday clinical workflows surfaces specific tensions that can undermine even the most deeply held humanistic ideals. Numerous forces compete with our ability—or even our desire—to deliver humanistic care. In the case of CP, one of the most pervasive is the shared conviction—among clinicians, patients, and caregivers alike—that data speak more objectively than lived experience. As our stakeholders cautioned, centering illness interpretations on digital signals risks reframing patients’ stories through the lens of machine-generated feedback. Anthropologists describe this phenomenon as an “idiom”: a culturally patterned mode of expression—verbal, behavioral, or somatic—through which distress or well-being is communicated in ways that reflect shared meaning based on local beliefs and values. Classic idioms of distress, such as “heavy heart” [72,73], “ataque de nervios” [74], or notions of hot-cold imbalance [75], function less as discrete biomedical signs and more as symbolic languages linking individual suffering to broader cultural meanings, social relationships, and moral concerns. If data become the dominant idiom through which we express or even conceptualize illness, we may lose the ability to recognize, convey, and intervene in the complex multitude of factors influencing health and illness.

These idiomatic shifts pose far graver threats than concerns about false alarms, opaque metrics, or data privacy—issues that, while critically important, are largely tractable and already receiving extensive scholarly and technical attention. By contrast, the greater danger lies in narrowing our collective capacity to perceive human realities by privileging quantifiable signals over the nuanced psychosocial factors that shape how illness is understood and experienced. From this perspective, dehumanized care represents not merely a violation of respect or rights, but a siphoning of human insight, potentially eroding clinicians’ curiosity and compassion as well as patients’ ability to articulate their own experiences.

Ironically, this outcome runs counter to CP’s original promise: to provide objective, reliable insights into complex disease states and, in doing so, bring us closer to the ground truths of human suffering. Data alone cannot constitute those truths. The critical question—one that our study helps illuminate—is how to integrate these deep data into care in ways that strengthen, rather than undermine, the humanistic foundations of clinical practice.

## Appendix

Multimedia Appendix 1 Accuracy, validity, and trustworthiness of computer perception tools.

Multimedia Appendix 2 Patient-specific relevance.

Multimedia Appendix 3 Utility and implementation challenges.

Multimedia Appendix 4 Regulation and governance of computer perception technologies.

Multimedia Appendix 5 Data privacy and protection.

Multimedia Appendix 6 Patient impacts and harms.

Multimedia Appendix 7 Philosophical critiques of computer perception.

## Abbreviations

AI: artificial intelligence
CP: computer perception
HIPAA: Health Insurance Portability and Accountability Act
REDCap: Research Electronic Data Capture

## Data Availability

The datasets presented in this article are not readily available because full datasets must remain unavailable to ensure deidentification of interview participants. Requests to access the datasets should be directed to KMKQ.

## References

[1] Akre S, Seok D, Douglas C, Aguilera A, Carini S, Dunn J, Hotopf M, Mohr DC, Bui AAT, Freimer NB (2024). Advancing digital sensing in mental health research. NPJ Digit Med.

[2] Insel TR (2017). Digital phenotyping: technology for a new science of behavior. JAMA.

[3] Babu M, Lautman Z, Lin X, Sobota MHB, Snyder MP (2024). Wearable devices: implications for precision medicine and the future of health care. Annu Rev Med.

[4] Liu JJ, Borsari B, Li Y, Liu SX, Gao Y, Xin X, Lou S, Jensen M, Garrido-Martin Diego, Verplaetse TL, Ash G, Zhang J, Girgenti MJ, Roberts W, Gerstein M (2024). Digital phenotyping from wearables using AI characterizes psychiatric disorders and identifies genetic associations. medRxiv. Published online December 18, 2024.

[5] Orsolini L, Fiorani M, Volpe U (2020). Digital phenotyping in bipolar disorder: which integration with clinical endophenotypes and biomarkers?. Int J Mol Sci.

[6] Bufano P, Laurino M, Said S, Tognetti A, Menicucci D (2023). Digital phenotyping for monitoring mental disorders: systematic review. J Med Internet Res.

[7] Mobbs D, Wise T, Suthana N, Guzmán Noah, Kriegeskorte N, Leibo JZ (2021). Promises and challenges of human computational ethology. Neuron.

[8] Torous J, Gershon A, Hays R, Onnela J, Baker JT (2019). Digital phenotyping for the busy psychiatrist: clinical implications and relevance. Psychiatric Annals.

[9] D'Alfonso S, Coghlan S, Schmidt S, Mangelsdorf S (2025). Ethical dimensions of digital phenotyping within the context of mental healthcare. Journal of Technology in Behavioral Science.

[10] Huckvale K, Venkatesh S, Christensen H (2019). Toward clinical digital phenotyping: a timely opportunity to consider purpose, quality, and safety. NPJ Digit Med.

[11] Martinez-Martin N, Insel TR, Dagum P, Greely HT, Cho MK (2018). Data mining for health: staking out the ethical territory of digital phenotyping. NPJ Digit Med.

[12] Mohr DC, Zhang M, Schueller SM (2017). Personal sensing: understanding mental health using ubiquitous sensors and machine learning. Annu Rev Clin Psychol.

[13] Martinez-Martin N, Greely HT, Cho MK (2021). Ethical development of digital phenotyping tools for mental health applications: Delphi study. JMIR Mhealth Uhealth.

[14] Mulvenna MD, Bond R, Delaney J, Dawoodbhoy FM, Boger J, Potts C, Turkington R (2021). Ethical issues in democratizing digital phenotypes and machine learning in the next generation of digital health technologies. Philos Technol.

[15] Shen FX, Silverman BC, Monette P, Kimble S, Rauch SL, Baker JT (2022). An ethics checklist for digital health research in psychiatry: viewpoint. J Med Internet Res.

[16] Kitson A, Marshall A, Bassett K, Zeitz K (2013). What are the core elements of patient-centred care? A narrative review and synthesis of the literature from health policy, medicine and nursing. J Adv Nurs.

[17] Todres L, Galvin KT, Holloway I (2009). The humanization of healthcare: a value framework for qualitative research. International Journal of Qualitative Studies on Health and Well-being.

[18] Watson J (2008). Nursing: The Philosophy and Science of Caring.

[19] (2010). Shared decision-making in mental health care. Substance Abuse and Mental Health Services Administration (SAMHSA).

[20] Insel TR (2018). Digital phenotyping: a global tool for psychiatry. World Psychiatry.

[21] Oudin A, Maatoug R, Bourla A, Ferreri F, Bonnot O, Millet B, Schoeller F, Mouchabac S, Adrien V (2023). Digital phenotyping: data-driven psychiatry to redefine mental health. J Med Internet Res.

[22] Kostick-Quenet K, Estep J, Blumenthal-Barby JS (2024). Ethical concerns for remote computer perception in cardiology: new stages for digital health technologies, artificial intelligence, and machine learning. Circ Cardiovasc Qual Outcomes.

[23] Pai A, Santiago R, Glantz N, Bevier W, Barua S, Sabharwal A, Kerr D (2024). Multimodal digital phenotyping of diet, physical activity, and glycemia in Hispanic/Latino adults with or at risk of type 2 diabetes. NPJ Digit Med.

[24] Provenza NR, Reddy S, Allam AK, Rajesh SV, Diab N, Reyes G, Caston RM, Katlowitz KA, Gandhi AD, Bechtold RA, Dang HQ, Najera RA, Giridharan N, Kabotyanski KE, Momin F, Hasen M, Banks GP, Mickey BJ, Kious BM, Shofty B, Hayden BY, Herron JA, Storch EA, Patel AB, Goodman WK, Sheth SA (2024). Disruption of neural periodicity predicts clinical response after deep brain stimulation for obsessive-compulsive disorder. Nat Med.

[25] Leaning IE, Ikani N, Savage HS, Leow A, Beckmann C, Ruhé Henricus G, Marquand AF (2024). From smartphone data to clinically relevant predictions: a systematic review of digital phenotyping methods in depression. Neurosci Biobehav Rev.

[26] Onnela lab. Harvard TH Chan School of Public Health.

[27] Picard RW (2000). Affective Computing.

[28] Barrett LF, Mesquita B, Gendron M (2011). Context in emotion perception. Curr Dir Psychol Sci.

[29] Alderman JE, Palmer J, Laws E, McCradden MD, Ordish J, Ghassemi M, Pfohl SR, Rostamzadeh N, Cole-Lewis H, Glocker B, Calvert M, Pollard TJ, Gill J, Gath J, Adebajo A, Beng J, Leung CH, Kuku S, Farmer L, Matin RN, Mateen BA, McKay F, Heller K, Karthikesalingam A, Treanor D, Mackintosh M, Oakden-Rayner L, Pearson R, Manrai AK, Myles P, Kumuthini J, Kapacee Z, Sebire NJ, Nazer LH, Seah J, Akbari A, Berman L, Gichoya JW, Righetto L, Samuel D, Wasswa W, Charalambides M, Arora A, Pujari S, Summers C, Sapey E, Wilkinson S, Thakker V, Denniston A, Liu X (2025). Tackling algorithmic bias and promoting transparency in health datasets: the STANDING Together consensus recommendations. Lancet Digit Health.

[30] Trustworthy and responsible AI resource center - AI risks and trustworthiness. National Institute of Standards and Technology (NIST).

[31] Adler DA, Stamatis CA, Meyerhoff J, Mohr DC, Wang F, Aranovich GJ, Sen S, Choudhury T (2024). Measuring algorithmic bias to analyze the reliability of AI tools that predict depression risk using smartphone sensed-behavioral data. Res Sq.

[32] Cross JL, Choma MA, Onofrey JA (2024). Bias in medical AI: implications for clinical decision-making. PLoS Digit Health.

[33] Khera R, Simon MA, Ross JS (2023). Automation bias and assistive AI: risk of harm from AI-driven clinical decision support. JAMA.

[34] Walsh CG, Chaudhry B, Dua P, Goodman KW, Kaplan B, Kavuluru R, Solomonides A, Subbian V (2020). Stigma, biomarkers, and algorithmic bias: recommendations for precision behavioral health with artificial intelligence. JAMIA Open.

[35] Shen FX, Baum ML, Martinez-Martin N, Miner AS, Abraham M, Brownstein CA, Cortez N, Evans BJ, Germine LT, Glahn DC, Grady C, Holm IA, Hurley EA, Kimble S, Lázaro-Muñoz Gabriel, Leary K, Marks M, Monette PJ, Onnela J, O'Rourke PP, Rauch SL, Shachar C, Sen S, Vahia I, Vassy JL, Baker JT, Bierer BE, Silverman BC (2024). Returning individual research results from digital phenotyping in psychiatry. Am J Bioeth.

[36] Tabassi E Artificial intelligence risk management framework (AI RMF 1.0). National Institute of Standards and Technology (NIST).

[37] European Union (2024). Regulation (EU) 2024/1689 of the European Parliament and of the Council of 13 June 2024 Laying down Harmonised Rules on Artificial Intelligence and Amending Regulations (EC) No 300/2008, (EU) No 167/2013, (EU) No 168/2013, (EU) 2018/858, (EU) 2018/1139 and (EU) 2019/2144 and Directives 2014/90/EU, (EU) 2016/797 and (EU) 2020/1828 (Artificial Intelligence Act). Official Journal of the European Union/European Union.

[38] Bahmani A (2022). Deep data and precision health. Inside Precision Medicine.

[39] Hurley ME, Sonig A, Herrington J, Storch EA, Lázaro-Muñoz G, Blumenthal-Barby J, Kostick-Quenet K (2024). Ethical considerations for integrating multimodal computer perception and neurotechnology. Front Hum Neurosci.

[40] Perez-Pozuelo I, Spathis D, Gifford-Moore J, Morley J, Cowls J (2021). Digital phenotyping and sensitive health data: implications for data governance. J Am Med Inform Assoc.

[41] Häuselmann A (2021). Fit for purpose? Affective computing meets EU data protection law. International Data Privacy Law.

[42] Lay W, Gasparini L, Siero W, Hughes EK (2024). A rapid review of the benefits and challenges of dynamic consent. Research Ethics.

[43] Brodkin E, Pallathra A (2021). Missing Each Other: How To Cultivate Meaningful Connections.

[44] Stroud AM, Curtis SH, Weir IB, Stout JJ, Barry BA, Bobo WV, Athreya AP, Sharp RR (2025). Physician perspectives on the potential benefits and risks of applying artificial intelligence in psychiatric medicine: qualitative study. JMIR Ment Health.

[45] Martani A, Starke G (2019). Personal responsibility for health: the impact of digitalisation. Journal of Medical Law and Ethics.

[46] Nghiem J, Adler DA, Estrin D, Livesey C, Choudhury T (2023). Understanding mental health clinicians' perceptions and concerns regarding using passive patient-generated health data for clinical decision-making: qualitative semistructured interview study. JMIR Form Res.

[47] Schmidt S, D'Alfonso S (2023). Clinician perspectives on how digital phenotyping can inform client treatment. Acta Psychol (Amst).

[48] Howard M (2021). Wearables, the marketplace and efficiency in healthcare: how will i know that you’re thinking of me?. Philos Technol.

[49] Warraich HJ, Califf RM, Krumholz HM (2018). The digital transformation of medicine can revitalize the patient-clinician relationship. NPJ Digit Med.

[50] Boyatzis R (1998). Transforming Qualitative Information: Thematic Analysis and Code Development.

[51] Braun V, Clarke V (2008). Using thematic analysis in psychology. Qualitative Research in Psychology.

[52] Kostick-Quenet KM, Hurley M, Herrington J, Storch EA (2025). Rethinking ethics for an era of trusted computational tools. Psychiatric Clinics of North America.

[53] Barrett LF, Adolphs R, Marsella S, Martinez AM, Pollak SD (2019). Emotional expressions reconsidered: challenges to inferring emotion from human facial movements. Psychol Sci Public Interest.

[54] Birk RH, Samuel G (2022). Digital phenotyping for mental health: reviewing the challenges of using data to monitor and predict mental health problems. Curr Psychiatry Rep.

[55] H Birk R, Samuel G (2020). Can digital data diagnose mental health problems? A sociological exploration of 'digital phenotyping'. Sociol Health Illn.

[56] Chen A (2017). A Neuroscientist explains the origins of emotions. The Verge.

[57] Le Mau T, Hoemann K, Lyons SH, Fugate JMB, Brown EN, Gendron M, Barrett LF (2021). Professional actors demonstrate variability, not stereotypical expressions, when portraying emotional states in photographs. Nat Commun.

[58] Barrett LF (2017). The theory of constructed emotion: an active inference account of interoception and categorization. Soc Cogn Affect Neurosci.

[59] Emanuel A, Eldar E (2023). Emotions as computations. Neurosci Biobehav Rev.

[60] Manta C, Patrick-Lake B, Goldsack JC (2020). Digital measures that matter to patients: a framework to guide the selection and development of digital measures of health. Digit Biomark.

[61] Kostick-Quenet KM, Herrington J, Storch EA (2024). Personalized roadmaps for returning results from digital phenotyping. Am J Bioeth.

[62] Sonig A, Deeney C, Hurley ME, Storch EA, Herrington J, Lázaro-Muñoz G, Zampella CJ, Tunc B, Parish-Morris J, Blumenthal-Barby J, Kostick-Quenet K (2024). What patients and caregivers want to know when consenting to the use of digital behavioral markers. NPP—Digit Psychiatry Neurosci.

[63] Maher NA, Senders JT, Hulsbergen AFC, Lamba N, Parker M, Onnela J, Bredenoord AL, Smith TR, Broekman MLD (2019). Passive data collection and use in healthcare: a systematic review of ethical issues. Int J Med Inform.

[64] O'Doherty KC, Christofides E, Yen J, Bentzen HB, Burke W, Hallowell N, Koenig BA, Willison DJ (2016). If you build it, they will come: unintended future uses of organised health data collections. BMC Med Ethics.

[65] Segura Anaya LH, Alsadoon A, Costadopoulos N, Prasad PWC (2018). Ethical implications of user perceptions of wearable devices. Sci Eng Ethics.

[66] Kreitmair KV, Cho MK, Magnus DC (2017). Consent and engagement, security, and authentic living using wearable and mobile health technology. Nat Biotechnol.

[67] Lee H, Lee U (2021). Dynamic consent for sensor-driven research.

[68] Rake EA, van Gelder MMHJ, Grim DC, Heeren B, Engelen LJLPG, van de Belt TH (2017). Personalized consent flow in contemporary data sharing for medical research: a viewpoint. Biomed Res Int.

[69] Basile MJ, Rubin E, Wilson ME, Polo J, Jacome SN, Brown SM, Heras La Calle G, Montori VM, Hajizadeh N (2021). Humanizing the ICU patient: a qualitative exploration of behaviors experienced by patients, caregivers, and ICU staff. Crit Care Explor.

[70] Busch IM, Moretti F, Travaini G, Wu AW, Rimondini M (2019). Humanization of care: key elements identified by patients, caregivers, and healthcare providers. a systematic review. Patient.

[71] Meneses-La-Riva ME, Suyo-Vega JA, Fernández-Bedoya VH (2021). Humanized care from the nurse-patient perspective in a hospital setting: a systematic review of experiences disclosed in Spanish and Portuguese scientific articles. Front Public Health.

[72] Berendt E, Tanta K (2011). The 'heart' of things: a conceptual metaphoric analysis of heart and related body parts in Thai, Japanese and English. Intercultural Communication Studies.

[73] Fabian K, Fannoh J, Washington GG, Geninyan WB, Nyachienga B, Cyrus G, Hallowanger JN, Beste J, Rao D, Wagenaar BH (2018). "My heart die in me": idioms of distress and the development of a screening tool for mental suffering in Southeast Liberia. Cult Med Psychiatry.

[74] Koydemir S, Essau C (2018). Anxiety and anxiety disorders in young people: a cross-cultural perspective. Understanding Uniqueness and Diversity in Child and Adolescent Mental Health.

[75] Vásquez-Londoño CA, Cubillos-Cuadrado L, Forero-Ozer A, Escobar-Espinosa P, Cubillos-López DO, Castaño-Betancur DF (2021). Principle of hot and cold and its clinical application in Latin American and Caribbean Medicines. Adv Exp Med Biol.

