# Supplementary material for: Stakeholder Perspectives on Humanistic Implementation of Computer Perception in Health Care: Qualitative Study
Source: JMIR Ment Health. 2026 Jan 5;13:e79182. doi: 10.2196/79182 (PMC12817037; doi:10.2196/79182)
Supplement: Multimedia Appendix 1 [file mental_v13i1e79182_app1.docx]

**Table 4. Accuracy, Validity and Trustworthiness of CP Tools**

**Data Quality Constraints and Confounds**

*Behavioral Variability & Consistency*

“**You have to control for things that are easily overlooked**, like the fact that **people don't wear a wearable all the time**. In fact, we know at the population level that about **half of the population stops wearing a wearable after four to five months**. **And, 90% stop wearing wearable after one yea**r. People who are **typically most compliant are typically young, healthy individuals**, especially people who are really into sports and exercise and stuff like that." (D_06)

"Because if everything is based on that, the model accuracy, then decision-making and everything. I**f you wear your watch loosely, you don't get good measurements.** And then the other parts are if you have a software update you miss. Software updates all the time, like **the robustness to all those external factors, which you're not controlling. I feel like it is the biggest set of problems in my opinion, operational problems.** So in research protocols you can manage to reduce these compounding variables, but once you deploy a system, this will become universal issues. And I feel like that will be a major bottleneck for widespread use, which is people can't get reliable data first." (D_18)

*Device Variability & Performance*

"Of course there's the **hardware element of this as well in terms of light sensitivity to darker skin**, all that kind of thing.” (ELPP_07)

“One challenge here is that these **commercial wearables don't tell you when the device is worn by the person. But, research grade devices do.**.. But when [validation] is done outside of a lab... in free living settings, that's actually when you have some of these big challenges.” (D_06)

**Algorithmic Bias**

*Generalizability*

“I remember reading that s**ome groups of people, when they speak, they don't move their body a lot,** so they don't have a lot of body language. **So for the type of machine that reads body language, I wouldn't really be effective with them**.” (P_11)

“Only 5% of the models in individualized clinical prediction models in psychiatry … get externally validated, which means that 95% are not generalizable.”  (C_09)

“If you use them **in a context where you have very little data, they will overfit.** And, now you have a true **problem with generalizability.**” (D_06)

*Non-diversity of training data sets & developers*

“But I feel like i**t could easily become something where it can marginalize a group and not give a certain group of people the right care because it misunderstands something or it's created by a certain race of people.** And then, it only applies to that certain race of people... Or gender might play a role in it. I**f it's created by men, then women may not be able to use it as efficiently.”** (P_07)

"I think there's **not very good data that we have in general on various ethnic populations**. So, I worry about that. I think we're making a lot of generalizations, and I know that the likelihood of some populations to be able to give data, just access to the population is limited. And so, **I worry about making leaps for whether it's a minority person or an age group that's hard to reach, older, younger, and anyone whose data is not there on..."** (C_08)

**"Who has access to this tool?**… That’s **going to create a bias in the data sets that we get** and the people that have access to this type of care, those are our potential concerns as well.” (ELPP_09)

"It's really **easy to get convenience samples that are primarily white and primarily high SES families**, the kinds of families who can take a day off work to come in for a research study. And **that is not the average child on the spectrum.** And so **I do have big concerns that computer vision folks will not sufficiently attend to the kinds of variables that could really impact things."** (C_19)

"I see a lot of stuff about **how poorly trained in terms of demographic groups, the facial affect technology is.** So maybe it works great on me because I'm a middle-aged white guy and there's lots of pictures of me. But if it thinks that Black faces are angry because it was trained on mug shots, and there's more people arrested who are Black, not because they commit more crimes, but because that's where the police are, then **the system is just eating its tail, creating all kinds of perpetuating of injustice... The data on which things like the facial computing ethics stuff is trained [is] going to have problems like tha**t." (D_08)

"**The issue was that was the training stage.** That there was hand labeling done beforehand and then the AI system will only do what it was trained to do in the first place. So **that bias issue is very much present in emotion based questions as well."** (ELPP_07)

*Off-label Use*

“…you c**an think of many different use cases**… [for] algorithms **for particular populations** or **for purposes for which they were not initially trained or intended**.” (ELPP_17)

*Variability in Symptom Expression*

"There are these **intangible aspects**… cultural, historical aspects of how we think about emotions that **don’t necessarily get reflected in these model building**…” (ELPP_17)

"My brain immediately goes to facial recognition technology that's used in criminal legal systems and how bias is so deeply baked into that. And I'm thinking about **the cultural constraints of affective expression and gesture.**.. I see a lot of first generation kids whose parents are refugees, or immigrants in their adulthood, and **I don't even know enough about how acculturation impacts affective expression. I think that's a concern for me..."** (C_13)

"How can you really control for the fact that **a smile might mean something in different contexts?** So not only within cultures, but across cultures…” (D_03)

**Construct Validity**

*Validity of Existing Diagnostic Categories and Assessments*

Uncertainty around Training Targets and Ground Truths

"A lot of **this tech kind of assumes that the diagnostic tools we have are cross-cultural.**.. But... Those categories **might not be completely true.**" (ELPP_01)

"You **might inadvertently...create...novel kinds of clusters** that **do not necessarily map onto our preexisting conceptual understandings** of categories" (ELPP_18)

"**What are we training to?** Are we training to a PHQ-9? Are we training to a Hamilton? Are we training to a clinical diagnosis or training the DSM? **It is not really clear to me. I think that's still been the biggest handicap for this field.**.." (ELPP_20)

"How do we account for even the fact that disorders that we are trying to detect discreetly and separately from each other, might actually be... Like problems of living, they might be **network problems rather than sort of distinct entities that can be detected and discerned."** (ELPP_01)

Uncertain Illness Ontologies

“…I think the biggest thing that scares me… we **don’t really have any objective markers**… we’re **kind of assuming that there’s an objective entity that we can find**, and that data’s going to be the answer…” (ELPP_16)

"...part of what I think **has been so problematic for mental health is the diagnostic scheme is A, doesn't really have a strong scientific basis using any kind of objective measures** and B, link in any way to treatment response or etiology, either one of those. So in some ways, it's a harder task than it is when you're looking for digital phenotyping in some other areas of medicine. And I guess the best example is using AI to read mammograms where you have a ground truth from having done a biopsy and which tumors are malignant an**d which are not. We just don't have that here. We don't have a biopsy, we don't have any solid footing that we're going to go up against, and that to me is the biggest challenge for the field."** (ELPP_20)

*External Cues May Not Reflect Internal Experience*

Behavioral Expressions

"I think in terms of the idea of reverse inference, so **you have an outward signal to detect an interior state..**. So from a clinical side of things, you have problems of diagnosis, is **what you're sensing from the outward. Does that have anything to do with what's going on inside a person?**... I think [**the assumption] that [there's] a connection between internal and external expression that's forever lasting and reliable is problematic,** I think." (ELPP_07)

Physiological (Bio-) Markers

“I think [physiological changes] can tell us something about the internal state of a patient... Is the heart rate spiking? What is heart rate variability? What is the skin conductance? How much are you sweating? You can tell all these things. [But] **I don't think you can use that data to then tell us something about what a person is feeling.**” (ELPP_01)

**Explainability**

If the model doesn't do what it's supposed to do, you can't open the box and say, "Ah, this is why it doesn't work." (D_06)
