# Supplementary material for: Stakeholder Perspectives on Humanistic Implementation of Computer Perception in Health Care: Qualitative Study
Source: JMIR Ment Health. 2026 Jan 5;13:e79182. doi: 10.2196/79182 (PMC12817037; doi:10.2196/79182)
Supplement: Multimedia Appendix 2 [file mental_v13i1e79182_app2.docx]

**Table 5. Patient-Specific Relevance**

**Accounting for Heterogeneity in Symptom Expression & Subjectivity**

*Capturing Subjective Meaning and Significance*

"I'm a very type A person. Go, go, go. To stay home for a while or don't get out of bed... even if I'm really stressed or having a bad day, I don't do that, because I'll always have a to-do list. Even if I'm having a bad day, I have to do these things. [**The system] might not realize sometimes that I'm overly stressed or upset, just because I'm still going on with my normal day.**" (P_17)

“I think [these technologies would be useless without subjective input] in the sense that there's no good healthcare without patient consent and input because it's about their health. **It's not about what we think is going on with their health, it's about their experience of it.** We should not use it if we are not going to take what the patient says into consideration." (C_12)

*Capturing Individual Heterogeneity*

"There's a **huge amount of individual variability**, and I think **one of the things we learned is this is harder than it looks.**" (ELPP_20)

“So, I think some people are very emotive and other people ... For example, for some people when they become anxious, they're nervous, they're fidgety, they're sweating, they're tremulous... But there are some people when they become anxious, they look disinterested... not tuned in... not paying attention. So **people will express those emotions differently. And would a computer be able to pick that up?"** (CG_01)

"If I'm sad, it's pretty obvious, if I'm pissed off, it's pretty obvious, but I know a lot of other people it would probably be harder. I**s [the algorithm] going to be able to pick up on the same exact signs it would for someone else?**" (P_19)

**Accounting for Context & Meaning**

*Sociocultural and Environmental Context*

"**That behavior has to be understood in context**… It **doesn't necessarily mean that the algorithm's going to work well in a very different context**. And that's an empirical question.” (D_03)

"[With wearable technologies,] **all that context gets lost.** And that **context is almost always informed by what is your life constellation?** What's your **cultural background?** Your **socioeconomic situation? Are you rural or urban?** And, and, and…" (D_20)

"Yeah, well, it's not [personalized medicine]. It **doesn't account for all really important things that happen in my day-to-day life. They don't appreciate my culture,** necessarily. They don't understand the sort of social issues I'm having, or they don't understand just these day-to-day things are going on in life." (ELPP_14)

*Personal Attributions of Meaning*

“It's **the context piece**, that you can see changes relative to context. And **context is often a very personal and private thing.**" (C_04)

*Temporal Contexts*

"Maybe **if something in particular happened that made you sad** for a period of time but **it's not permanent,** then I know that **I wouldn't want that to be taken out of proportions and maybe it would become a 'thing', even if I didn't want it to, it was just something temporary...**stuff like actual emotions, I don't know... **sometimes those change really fast and I feel different... I don't know if it would be able to pick up on that as well as it thinks it can.**" (P_14)

"I think for the most part I'd be fine with [passive data collection], but **if it's constantly picking up on the five minutes that I just didn't get exactly what I wanted** and i**t would just be like,** 'Oh my gosh, you're not feeling okay, **you are possibly depressed**,' and it's like, '**No, it was just a five minute thing**.' I feel like it **could eventually get to the point where it's annoying**..." (P_19)
