# Supplementary material for: Stakeholder Perspectives on Humanistic Implementation of Computer Perception in Health Care: Qualitative Study
Source: JMIR Ment Health. 2026 Jan 5;13:e79182. doi: 10.2196/79182 (PMC12817037; doi:10.2196/79182)
Supplement: Multimedia Appendix 3 [file mental_v13i1e79182_app3.docx]

**Table 6. Utility and Implementation Challenges**

**Role of CP in Clinical Care**

*Tech Overreliance*

Uncertainty around Training Targets and Ground Truths

"And there is research about **clinicians relying too much on the decision support and not questioning it.** A bit like somebody on a conveyor looking at a conveyor belt in a manufacturing factory where there was AI sensing for defects. **In the end they're not doing anything. They're like someone sitting in a driverless car. They're not really a human in the loop...**  Who's making life changing decisions or life affecting decisions?" (ELPP_04

"And so I think what you're seeing is that t**here may be a distributional gulf that increases in the quality of hyper users with maintaining the domain expertise and low intensity users who become in fact fully reliant on the systems.**" (D_19)

"In the worst case scenario, I was thinking about how would I feel about this if I was a first year therapist in grad school doing training, and I wonder... It could be that it's useful because it's like, 'Oh, here's some things to consider.' But it also **could be that it makes someone doubt their own burgeoning clinical judgments or, 'Well, the computer said this, so I guess I must have missed that and let's go with that.'"** (C_15)

Automation Bias

"If an AI just **presents one recommendation,** then the **user could be prone to automation bias.** Automation bias being the overreliance or the **complacency of just accepting whatever the AI says**, without... And are we as rigorous in looking at the data when there's AI assisting?" (D_15)

"If there's some sort of problem with [the inferences] and you're just sort of taking it as like, "Oh, okay, this is what it says", **it could make you make the wrong sorts of decisions. Or again, maybe make you think... 'Maybe they *do* have a personality thing, I wasn't detecting that, but maybe they do.' And maybe you just sort of muddle the picture up a little bit in the worst case scenario**." (C_15)

Loss of Human/Relational Interpretation

"AI is good at picking out patterns… but **quite bad at understanding**  **what that pattern means in the context of the individual**… it’s still really about the clinician and their patient.” (C_04)

*Managing Risk and Liability*

"Then, obviously, the false negative too, right? I mean, as a clinician, **I worry about, say, there were an adverse outcome,** following what looked like normal readings from the technology. **Does that put me in the hook for litigations and so on?**" (C_16)

"And I think **other risks are also the liability**. There's a reason why, when we automate depression screeners, we exclude the question that asked about suicidality, because **there's an obligation to then take that seriously... what would happen if the system isn't able to respond in the moment to that?"** (C_13)

**Barriers to Utility**

*Interpretability*

Consistency of Meaning

"If you're a company and your whole business model is based on sending out alerts when somebody's having a psychiatric episode, **it has to be based on a real finding, on a real phenomenon...** If you roll it out at scale, **it has to be something that's repeatable**. [But] **making sense of the data is a lot more difficult than people realize... It's harder to extract the gold than what people really think**." (D_06)

"People don't understand. **Let's say I make a decision for a patient on software version 1.0.3.1, and then somewhere in the background it got updated. Now, the algorithm says I should have made a different decision, or it shifts the probabilities in my differential diagnosis differently.... Now what do I do in this case? There's a legal issue, there is a care issue.** It's not like a thermometer, which is going to be thermometer yesterday and today the same way. **An algorithm** doesn't behave that way; it **has a fluidity... which we do not know... how to really deal with it.”**  (D_18)

"Even if you trust the heart rate variability numbers that come out of this actigraph that you're using, then what do they correlate to? **Do they always mean this? Do they always mean that? How do we know that it means that in this population or that population?** What is the practical effect really?" (C_20)

Confirmation Bias

"In radiology specifically and diagnostic imaging fields, **the clinicians' ability is they're experts in interpreting images and being able to communicate that knowledge.**.. We know that clinicians like radiologists have training in being able to identify whether there's a pulmonary nodule or not, **but I think we need to recognize that the introduction of these (CP) models, if not designed carefully, can lead to confirmation bias, which is looking at things in the image to confirm what you want to see in the image.** [Conversely], **if you're someone, you do not consider yourself at an expert level, let's say in training, you will be in a cognitive dissonance stage where you are questioning yourself and believing that the model is somehow truthful,** and that can lead to more inefficiencies than efficiency if this happens a lot." (ELPP_10)

Data Overload

"If I was recording all my weight and GSR and if I was recording EEG during sleep, pulse oximetry at home and blood pressure, and... uploading that into some electronic care record, will my GP feel responsible to read that and interpret that? **That kind of burden of lots and lots of information and data.. The more signals that we're providing, then they need to be interpreted. But is it by a computer, by a human or both?**" (D_15)

"I think that what will probably happen on the clinical side is that people will get into wearables and these things, patients themselves. And, it **may be difficult for clinicians to make sense of the data or these metrics** because it might be a different device, it could be something very complicated. So, I could see on the clinician side that **clinicians might feel overwhelmed**." (D_06)

*Risk of Non-Actionable Metrics*

"A six minute walk test **might seem like a meaningful measure to a patient** going into a clinical evaluation for how well they ambulate, but we've seen over the course of some decades that **it's not actually meaningful.**" (ELPP_19)

"...even if you trust the heart rate variability numbers that come out of this actigraph that you're using, then what do they correlate to? Do they always mean this? Do they always mean that? How do we know that it means that in this population or that population? **What is the practical effect really?"** (C_20)

*False Alarms / Positives*

"Having the measure is never a problem. It's **how it gets used and who it's provided to, that can create the problem.** And so, people can be falsely alarmed by these kind of things." (D_13)
