# Supplementary material for: Stakeholder Perspectives on Humanistic Implementation of Computer Perception in Health Care: Qualitative Study
Source: JMIR Ment Health. 2026 Jan 5;13:e79182. doi: 10.2196/79182 (PMC12817037; doi:10.2196/79182)
Supplement: Multimedia Appendix 4 [file mental_v13i1e79182_app4.docx]

**Table 7. Regulation & Governance of CP Technologies**

**Acceptability & Uptake Requires Regulation**

*Subject to Existing Clinical Use Regulations*

"One thing that shifts the calculus a lot is that the **technologies that are going to get used in a clinical setting... will be subject to more regulation than those that are [direct-to-consumer]..**. I think the regulation, as much as people hate regulation, I think is a ...key factor here because... whether you call it consumer use or patient use, [what] **you're engaged in essentially medical interactions or interventions.** So I think that's really different, the regulation part." (ELPP_12)

"Many of the companies that are creating these technologies are **not necessarily aware of or are maybe actively trying to skirt some of the existing regulations and they think simply because it's a very novel technology that certain regulations don't apply** or they're outdated and therefore, won't affect them. Equally, some clinician researchers in the field are maybe **proceeding without necessarily contemplating how privacy law might interact with these technologies** or laws related to telecommunication technologies and so on." (ELPP_15)

*Need for Implementation Guidelines*

"You **need governance pathways implemented with trust**, **where the boundaries are clearly defined and operationalized.** **You can't simply ask the clinicians to use these tools out of the blue without these guidelines**." (C_09)

**Unclear / Insufficient Regulatory Frameworks**

*Regulatory Gray Zone*

"So, one challenge that's coming up in the mental health field is where apps are pitched as being about, wellbeing rather than necessarily treating a mental health condition. An app might be doing very similar things, tracking the same data, continuous monitoring, and potentially suggesting certain things that might be going on. But **because it's built in terms of wellness, then that takes it outside of the governance regime of medical devices."** (ELPP_15)

"Yeah, I mean, I think one important question to ask is, **is this thing offering me medical advice? Or is this thing practicing medicine?** And if the answer is yes, then it should have the requisite regulatory approvals to do so. And **if not, then it should be very, very clear that the clinician is in the loop..**.I think **that's where any company maybe could get in trouble in that gray area.** There is a pretty bright line between practicing medicine and not. **You want to be very clear with your users, and as a user you want to be very clear what's happening here."** (D_09)

*Oversight for Passive & Contextual Data Collection*

"I think the second, so one is **regulation of contextual data in addition to medical data.** ... The problem is those are often not being mediated as medical applications. Those are **commercial applications that are collecting that data with little regulatory oversight.** So I'm very concerned about that." (D_19)

**Responsibility for Ethical Tech Development and Compliance**

*Balancing Innovation with Regulation*

"**Feasibility, it's super demanding. Something extra. [**Many developers are] saying 'We're already taking so many things into account and we're pretty willing to take ethics into account,'... But then **you come up with this [ethical] framework** which is... super specific and really demanding... And so they [are] **not so keen on taking on that extra work...**" (ELPP_08)

*Deploying Proprietary vs. Open-Source Algorithms*

"I would say that **my concern,** looking around my [the tech development] industry**, is the blase attitudes that folks have when deploying proprietary algorithms for clinical use cases.** Because **we don't want our care system or clinical research practices to be in a place where they are dependent on a private algorithm to give access to care.** We've made that mistake in the past... being dependent on organizations like that... and so I wish that there were more checks and balances, or an awareness on that." (D_13)

*Unclear Liability*

"**Are [patients] responsible** for seeing this alert and communicating? Is it internet-connected and alerting the clinical team? If [so], is it only for research purposes, and therefore you [the patient] actually have to pick up the phone and call your doctor? **Or does it create this false safety net that you think that your doctor is monitoring you because you have this wearable on you, but actually there's nothing that is alerting the doctor** to act on your behalf? So, I think **making sure that it's an inclusive approach and communicating to a patient how it works and what they're responsible for, and what the clinical team is responsible for, would be incredibly important.**" (ELPP_19)

"**What's different here is the passive track**... [that] tracking happening in someone's personal and private life **outside of a clinical encounter**... I think there are **lots of open questions there about what the clinical relationship looks like** when that's happening, and **where the duty of care extends to**." (ELPP_16)

**Need for Stakeholder Involvement**

*Accounting for Lived Experience*

"Any project that's **trying to develop a technology for clinical care should absolutely have... at least one person, if not multiple people, with lived experience.** If [it's] for depression and anxiety, well, you need people who've experienced depression and anxiety **involved as co-leads on the project**. ...A lot of tech companies and organizations are developing technologies because there's money in it, not necessarily because they actually want to solve a problem or care about the end, **the person who it's going to impact. [Those] people have alternative understandings of their symptomology, which aren't medical understandings. And they're valuable... Those kinds of understandings need to be incorporated into the technologies.**" (ELPP_16)

*Need for Interdisciplinary Collaboration*

"I'm working on teams with machine learning experts, with clinicians, with clinician researchers, with stakeholder groups, et cetera... general population consumers. One of the issues... is you get people who are really well-versed in this area. You get people who know the technology and are quite protective of their understanding of the technology. I see it happen all the time: You **get in meetings and the person who talks really well about the technology basically runs the show**." (ELPP_14)

*Understanding How & Whether CP Matters*

"**What is the clinical meaningfulness, even if we know that, 'Yeah, this is accurate.**' Those are some of the questions that come to my mind." (C_20)

"I think that is a con as well... that **a lot of this tech is developed without really involving users as to what kind of features are meaningful to them.** As far as I know, they're very rarely involved in this kind of research. **What actually matters to people?** What do they want monitored? Do they want these technologies? **Does constant monitoring in a sense,** make you feel better? What if you have health anxiety? ...Maybe you actually don't need... Maybe that is actually detrimental to you.." (ELPP_01)
