# Supplementary material for: Stakeholder Perspectives on Humanistic Implementation of Computer Perception in Health Care: Qualitative Study
Source: JMIR Ment Health. 2026 Jan 5;13:e79182. doi: 10.2196/79182 (PMC12817037; doi:10.2196/79182)
Supplement: Multimedia Appendix 5 [file mental_v13i1e79182_app5.docx]

**Table 8. Data Privacy and Protection**

**Consent and Awareness**

*Unwanted / Unintended Disclosure*

"The biggest concern is just privacy... **it is going to collect stuff that you may not want another person to know, and if you don't realize all of that or you're not thinking all that through, it could be upsetting.**..I mean, most of us have, our phones are [already] tracking us with all kinds of health data app." (C_12)

"[It's] **a big thing on your privacy**, then you're sharing a lot so **there might be times where, 'Oh, I don't really want to share things right now**.'" (P_16)

“**People have a right to privacy, and there’s also just a limiting of agency in what you choose to share with your doctor or your provider.** Even **in clinical relationships or therapeutic relationships, the patient always has agency to talk or not talk about whatever they want, and share or disclose whatever they want. And that is also a meaningful process to allow patients to have agency in sharing that.**” (C_13)

“Well that **seems clinically very awkward. And uncomfortable.** I think **if a patient doesn’t want to share something, there’s probably a reason. And does that then put both the patient and the clinician in a weird position?** It’s almost like when a parent tells you something about the kid but then says, don’t tell the kid that I told you this. … **If it was sort of non-voluntarily shared, that seems kind of not ideal.**” (C_15)

"**People may say, 'Okay, I never consented my data to be screened for that…**'” (C_09)

"It's a little **bit creepy... Would I want all that information?** Generally, [we] have an expectation in life that certain things we can keep to ourselves and certain things are apparent to others. And **the whole idea behind this technology is, 'Hey, you can tell us things without actually telling us things. So we'll know things about you just by collecting this multitude of information about your facial expressions or your heart rate or things like that.' So there's that little bit of it that feels a little bit creepy."** (C_14)

*Appropriateness*

"I think there's a question about **whether we should be doing that**, **whether that is an appropriate way to integrate these kinds of tools into clinical care** when they're going to be tracking people in their homes, in their private lives, **revealing information that is sensitive and completely not health-related to their clinician.**.." (ELPP_16)

“There is **quite a distinct ickiness factor to being able to look that deeply into someone's personal life**. As a clinician, when I've had the opportunity to work with data like that or look at data like that, **even when it's deidentified, it's quite uncomfortable.**" (C_04)

*Coercion*

"We almost **moved into this very coercive mode where** the alternatives, if you don't have these digital tools in some of the austere settings we're doing work right now, **the alternative is basically nothing.** So, what you're saying is like, **'I will give you this care or do this research and we will give you all of these things, but you have to give up all of your data.'** There is no alternative here." (C_07)

"It's like, '**Give us all this information and we'll provide you really good healthcare, but you can opt out. But just understand that we might not give you the healthcare that you need.' There's not actually options there."** (ELPP_14)

*Patient Communication & Understanding*

"The other thing is about consent... **how would we set up consent for this information to be communicated to us?** Especially with this passive data collection..." (C_04)

"And of course, it **might differ for what kind of disease or condition you're experiencing**, but I think in lots of cases **people are quite vulnerable and it's really questionable whether they truly understand what the technology is doing, how it functions, what kind of tools it's using, what the end purposes is.** And that could then take on specific forms that are actually not beneficial for the patient." (ELPP_08)

*Perceived vs. Actual Data Sensitivity*

"Most **people are not concerned about something like accelerometer data or things of that sort. [But] All those studies have demonstrated that you *can* identify the person**... **The perceived risk of accelerometer data is extremely low. But, that's only *perceived* risk. The actual risk is actually higher for that category.** For GPS, people are much more aware of that risk. But, again, **if you have a Facebook account, you already incur that risk of privacy loss,** specifically related to where you are... The resolution might be plus minus 100 meters, 200 meters, as opposed to GPS which is plus minus three or four meters. But, still, there's location there." (D_06)

*Patient Right Not to Know*

"But if they **learn something about themselves they didn't want to know, that's hard.**" (ELPP_03)

"Yeah, exactly. That's definitely an ethical dilemma. We have gene tests that do the same thing now. You can test someone to see if they have a gene for Alzheimer's disease or Huntington's disease before they ever developed symptoms of that and before you would even know. I think it's a similar thing, but there **are ethical implications to knowing that information and whether or not that information gets shared with patients and whether they want to know that information.**" (C_14)

**Secondary Use and Misuses**

*Uncertain Secondary Uses*

"I have no problem whatsoever using an app where I'm supposed to be clicking: 'I feel like I'm an eight out of 10 in terms of anxiety today' or letting it track my heart rate, letting it track my gait... **The GPS stuff seems like it starts to cross some lines too and I would just have a lot of concerns about how that data could be used....**" (CG_12)

*Discrimination Potential*

"**Who knows how corporations may use that [CP data] in different ways to flag or discriminate against your child later on based on this kind of information, so that would be a concern, especially in the workplace.**" (CG_02)

*Lack of Existing Data Protections*

"Companies are sharing and using data for purposes other than why the data was initially collected... **If you really don't want your data used for other purposes, then there's not a lot of protections that exist right now."** (C_14)

"**The more I learn about how the regulation works, what law governs what data can move and who can share what with [who]**... Every entity can be fully lawful, law-abiding and then still... **the framework, regulatory framework leaves holes, leaks for people to exploit**." (D_18)

*Data Ownership & Control*

"**This information [should be] kept exclusively for the treatment purposes of the pathology the patient comes in to treat...** patient data **should be the property of the patient** and **only used within the realm that they have agreed upon.**" (C_10)

“We are selling out our healthcare data to groups like Google, Amazon, etc. **We are setting up our healthcare systems in a situation that healthcare professionals can no longer control their own data**…” (ELPP_14)

*Commercial Influence*

"I think that as healthcare systems begin to act like corporations, the **corporatization of healthcare data will mean that monetizing facial expression data, location data, mood data, wearables, is going to be an incredible risk for healthcare systems.** So I'm very worried that healthcare systems have to be reinforced socially as having a **fiduciary duty to patients as patients, rather than... to stakeholders**." (D_19)

"If my doctor had told me a year ago, I really want you to sleep better, so try this out," and I did it for a while and I thought it was only my doctor looking at it. [That becomes] very different, if I **could imagine ads being shown to me based on maybe Instagram somehow has access to my Oura data** on my phone and **can see that I'm acting manic, and they try and sell me specific things because of how my heart has behaved in the last week.** The crossing of the streams... thinking about what the potential benefits and harms are, that'd be the thing that would concern me... **The corporate environments, where the machinery that turns data into money, the people in those rooms, they don't live by [the] same rules [as us]**." (D_08)

**Monitoring & Surveillance**

*Can Exacerbate Patient Distrust*

"I think that some of the risks that I'm concerned about are how it would negatively impact the public's stigma towards mental health care. I think that it could dissuade people from engaging in that. **It feels like policing, which is not great, and I think for marginalized communities, extra not great...** I haven't really seen how this is applicable to kids, but **people already perceive me as policing them because I'm a mandated reporter,** so there's that." (C_13)

"And a reasonable response might be, 'Well, this is **just another form of surveillance. Why do they do this?**' And especially, again, i**n people who might be vulnerable with mental health issues, the last thing you want to do is give someone something that they think is increasing surveillance if they're having some issues around surveillance**." (ELPP_14)
