# Supplementary material for: Stakeholder Perspectives on Humanistic Implementation of Computer Perception in Health Care: Qualitative Study
Source: JMIR Ment Health. 2026 Jan 5;13:e79182. doi: 10.2196/79182 (PMC12817037; doi:10.2196/79182)
Supplement: Multimedia Appendix 6 [file mental_v13i1e79182_app6.docx]

**Table 9. Patient Impacts and Harms**

**Harms Due to Inaccurate or Premature Diagnoses**

“I guess what’s scary about it is I don’t know **what happens when the algorithm gets it wrong** and somebody is *not* going into a depressive episode and they’re called in and they’re like, I’m fine. I don’t need any intervention. Or **how will they feel about that**?" (D_10)

"**What if, based solely on this computer technology, some kind of medication is prescribed and maybe the computer got it wrong,** or just something wasn't right, and then they were taking the wrong kind of medicine? That, I could see being an issue." (CG_20)

“At this stage, I’m not really sure there are many (CP models) that are clinically validated outside of specific controlled environments. And even if they were to be reliable in identifying things that do seem to correlate with what we know as depression or schizophrenia or whatever, **there are going to be instances where they get it wrong and then decisions are going to be made that have very, very serious implications for people.**” (ELPP_16)

**Diminished Human Connection in Healthcare**

“What we tend to forget or maybe overlook is quite often in mental health, the **human contact is really, really important**. So **if we’re doing anything that, in fact, isolates people from their support networks or makes them think that they should be perhaps using this technology instead of seeking assistance with their human networks**… that can be **really damaging and really dangerous for people with mental health issues... t**he **care relationship is really important in healthcare**, incredibly important in mental health and psychiatric health. That relationship, there’s that **interdependent relationship. We rely on each other**." (ELPP_14)

“...I think t**here's relationships that are formed with your doctor, and I just don't see that computers can ever replace that.** I hope they don't... but **if we get to a place where they do, I think that mental health in kids and adults will be worse off altogether because of the lack of value on relationships,** not just with your healthcare provider, but in general.” (CG_17)

“I think **there's a lot lost in terms of the physician or therapist‐patient relationship...** So it used to be we would sit face to face and talk. Now... there's a computer there that's constantly being typed on and there's not a lot of eye contact... **that does concern me, that we're going to be more and more drawn into a virtual meta world rather than being in the real world with each other.** So I would want to see these used in conjunction with real human interactions...” (C_03)

**Responsibility Shifts and "Empowerment" Pitfalls**

*Patient Disempowerment*

“We hear a lot of rhetoric, which is actually, I think, quite damaging. … this notion of empowerment [with digital health technologies]. … Really, what’s actually happening is **we’re pushing responsibility away from the state onto individuals**. People who are advantaged might be able to take charge and respond to that. A lot of people who need care aren’t in the position to do that. So what we end up doing is we push blame. **Rather than making people empowered, we actually make them responsible, and then we blame them for their health inequalities and their health issues...** And then you get individuals who don’t see improvements. It can **actually then worsen their self-image** where it’s like, ‘Oh, gee, I’ve got all this support and I still can’t sort it out. Everyone else is. **I’ve got this tech. Why can’t I get this? I’m empowered.** This is now my responsibility. **Why can’t I do something about this?’**” (ELPP_14)

“I mean, **I feel apprehensive about the term empowerment** because immediately I ask, well, **who's saying someone is going to be empowered? Is it the person describing their own situation?** Or is it someone projecting that onto others? Empowerment is a word that seems to have been floating around mental health policy and probably other health policies since the 1990s... And was **part of a much broader trend towards transitioning from seeing a person as a patient to seeing them as a consumer of services**... And I think empowerment is something that has just continued through mental health services and other areas of health, and is definitely being taken on by the technology sector, because it is exactly **seen as an individualizing responsibility passed on to the person who's reframed as... a platform user. I think that narrative can hide a lot of those things...**" (ELPP_15)

*Deferral to Technology*

"[The CP] system **might be doing it for them**... 'I **don't have to worry about that because my iPhone is doing it for me**' or whatever." (C_15)

**Access Inequities and Disproportionate Burdens**

*Inequitable Access to Benefits*

“There might be a large proportion of the population that benefits. … But we need to look at the boundary cases and the marginalized populations and the vulnerable and the extreme harm that we can do to them. … **Are we systemizing disadvantage? Is there systemic forms of discrimination? And with a lot of technology at the moment, the answer looks like it’s going to be, yes, there is.** There’s systemic disadvantage. Who’s missing out? Okay, our aboriginal populations tend to be, our vulnerable groups, people who experience poverty, people who experience homelessness, our culturally diverse populations, our populations, LGBTQI+ plus populations. … So **these things really stack up** and become problematic.” (ELPP_14)

*Surveillance and Policing*

“We often forget that, certainly around psychiatric health, but **certainly around a lot of our marginalized vulnerable communities, they have a real fear of surveillance and interference. …** We’ve surveilled them and interfered in their lives so incredibly … So **if you give them a technology … a reasonable response might be, ‘Well, this is just another form of surveillance.** Why do they do this?’” (ELPP_14)

**"Big Brother** is what I think of right away, and the **potential [data] misuse.**” (CG_16)

"[Based on what] these technologies could determine or perceive... w**ould there then be automated [action]?** Just the implications of that. I think that that is something that I always have concerns about... **We're developing this technology for a hypothetical good in mind, but that technology could then be... leveraged into something that's more literal policing,** like would the police be dispatched if there's a mental health crisis? And knowing that **police involvement during mental health crises always goes badly for the folks who are experiencing mental health crises**, that's definitely a risk." (C_13)

“In terms of things to worry about, I think I probably worry about the idea of, really, persistent surveillance … **that’s problematic for me that they turn into a tool of surveillance**..." (ELPP_09)

*Harmful Biases*

"Who has access to this tool?... To the extent that that’s **going to create a bias in the data sets that we get and the people that have access to this type of care, those are our potential concerns as well.**” (ELPP_09)

*Pressured Consent*

“That’s an inherent problem in all method of tracking or surveillance. I always worry about, **there’s people who inherently are less empowered than others in virtue of their social status** … might feel less comfortable saying no to something like this, and it might actually exacerbate their mental health... **Do they feel this obligation to really do this quite invasive thing that might make them feel less at ease than they already are?**” (ELPP_12)

*Involuntary Monitoring and Detention*

“So, the idea of introducing these tools that **might be used at scale to diagnose people and then to make treatment decisions for people who might be subject to an involuntary detention** order is scary, I think, where we are not actually sure that the tools are tracking anything. … **there are going to be instances where they get it wrong and then decisions are going to be made that have very, very serious implications for people, for their rights, for their treatment** …” (ELPP_16).

**Threats to Privacy and Self-Determination**

*Discrimination*

"If [CP data could] I don't know how to describe it, work as a bias. He's applying to college right now. **Is there a potential that something could exclude him from a scholarship or an activity?**" (CG_19)

“...thinking from a bad movie plot, could it be that someone’s like, well, **we’re going to eradicate all gender dysphoria, so anyone who’s gender, this gets spiked on their data, we’re pulling all them** and we’re going to whatever, whatever. That sort of thing. I don’t know. I’m sure for anything that there’s good things about, there are ways that not good people can make it bad.” (C_15)

"Identity theft… that there would be **a clone of my daughter somewhere**…” (CG_16)

*Psychological Burdens & Preoccupation*

"There is a well-known phenomenon [in] diabetes called diabetes distress … **continuously being exposed to your data … can actually lead to** … your glucose is better managed, but now **you are psychologically stressed out** about it so it has other consequences. So you **may be solving one problem and creating another one.**” (D_18)

"Maybe [my daughter] **would hyper-focus on the results**, like how people say don’t Google your results because your eye itches and now you have cancer behind your optic nerve… So that might be something because she does **tend to hyper-focus and obsess sometimes**. So that would be a concern.” (CG_05)

**“I don’t want [my daughter] to have to think about [data collection] on a regular basis** and know that, ‘Oh my gosh, **everything I’m feeling, everything I’m doing is being recorded somewhere.’ I wouldn’t want it to affect her.**” (CG_20)

“**Folks would argue, ‘Yeah, but it’s all just more information**. You can do with it what you will.’ **But that’s not psychologically how people work.** If you tell me you have an increased risk of this, it’s not just information. Now, I’m someone who has this. And how do I go about my day? So **I really worry about returning ‘insights,**’ A, before they’re super validated, but also how you return them.” (ELPP_12)

"The manner in which you deploy and develop products around those algorithms. My primary care doctor is annoyed at how often her **patients will reach out to her and say, 'Oh my God, my Apple Watch told me this.'** And that is a**n example of the clinician being berated with data that is a distraction to them and their efforts to provide good care to their patients.**" (D_13)

*Altered Self-Perception and Behavior*

“[Information about my feelings or behaviors being automatically collected in the background] **might make me kind of self-conscious** about it and I don’t know, it **might affect the way I act because if I’m trying to act a certain way to act for these devices, it might be changing me, which I don’t know if that’s a good thing**.” (P_14)

“So **if we’re recording your voice...is it going to affect how you interact with other people because it’s being recorded a certain way**? **What are the sequelae for your social and personal life going to be?**… There’s the people who can always say, ‘Well, I don’t want to do this anymore,’ and then there’s those who feel beholden to it and will continue to trudge along. So, yeah, sure, **that’s something that I do worry about** with that kind of application.” (ELPP_12)

“I think maybe even the emotions could be very out of context, and if it were in context, that would be way too invasive. So that that's an issue. Monitoring location, I really don't like that. Again, anything that would have to do with food intake and even exercise. **Just things that if she's aware is being gathered, I don't know if it would be honest [accurate], because I think it would change the way that she would act.” (**CG_07)

“We humans lie. I don’t know how that would be taken into account… **If you might sense that, ‘Oh, all of this has been picked up. Do I have to start acting a certain way? Do I need to start saying certain things?** Is my response truthful? Is it not?' And so forth.” (CG_16)

*Internalization of CP Inferences*

“But then I worry, my bigger concern is then taking that and then relaying back to the patients or the users … **It’s one thing to see raw data, but once you do the insight thing and you put the stamp of scientific approval on it, that means a lot to people**. And so they see that and they say, ‘Oh my God, I’m depressed.’ Or, ‘Oh my God, I have a sleep disorder,’ or whatever. … And then they **potentially self-incorporate that into their identities**.” (ELPP_12)

*Gamification Impacts on Addictive Behavior*

“Yeah, it depends a little bit again on our understanding of vulnerability and whether or not we also have a sense of who might be more susceptible to these types of, I would say, habit-forming technology. … But **let’s say they’re at the moment engineered to habitualizing people to using this technology. Even this would be problematic in the consumer domain.** My understanding is that at Stanford University, you can actually take an engineering course where you can learn how to make apps more... addictive, if you will.” (ELPP_18)

*Feedback Impacts on Autonomy*

“Should people generally consent to getting these kinds of prompts and recommendations that guide their behavior in daily life with the promise that they feel better after a while, or **does this amount to a paternalistic nudging approach where self-autonomy is compromised?**” (ELPP_18)

**Epistemic Injustice and Deprioritization of Patient Voices**

“Yes, we should respect the lived experience, the client’s subjective perspective, but **what were to happen if there was this hypothetical oracle, this oracular system that was almost perfect, and it spat out something that was contra the patient simply because the patient wasn’t fully aware of something?** … **going against the patient may create some other ripple negative repercussions.**” (ELPP_13)

“At a time in history where I suppose mental health and disability activists and people with that lived experience are trying to push back to some extent on expert knowledge as the basis for decision-making about policies and programming that affects people with mental health conditions... in some ways, **computational technologies like digital phenotyping are really promoting a kind of expert monitoring, that has the potential to make claims about what is happening with a person’s internal state that may be quite different from what that person themselves is experiencing...** that **might have an impact on then how that person self perceives or how others perceive them.**” (ELPP_15)

"**I wouldn't want a provider to be using that data to exclude what I'm telling them** there in that moment... [about something] you're very concerned [about]... Because **I think that a parent's observations should be every bit as important as the data, if not more** depending on what exactly it is we're talking about.”(CG_12)

"One of my concerns would be how it would affect the doctor relying on, like if you were advocating for your child or if you were advocating for yourself, **I would worry that they would place more emphasis on the technology, the information, than they would on [your concerns]...** And I realize some people aren't good at communicating, but then sometimes we have these mom instincts that are stronger, I feel like, than anything really.” (CG_17)

**Overemphasis on Self-Optimization**

“I understand self-optimization, that's just some people's personalities... If one or two people want to do that, that's fine. **My concern is that it becomes more culturally acceptable to the point where we'll be starting to expect this of people the way no one's allowed to not have a smartphone today, you can't exist in society and not have a smartphone**, it's just absurd, **to get to a point where we're just expecting of people to have that level of self-surveillance.** And I think that there's some losses there with respect to how we exist in the world, this constant quantifying of the self... **Using it for a clear, clinical indication, versus using it for a project of self-optimization, I think that's a distinction that probably also maps onto the difference between the direct-to-consumer and the medical setting**.” (ELPP_12)
