# Supplementary material for: Stakeholder Perspectives on Humanistic Implementation of Computer Perception in Health Care: Qualitative Study
Source: JMIR Ment Health. 2026 Jan 5;13:e79182. doi: 10.2196/79182 (PMC12817037; doi:10.2196/79182)
Supplement: Multimedia Appendix 7 [file mental_v13i1e79182_app7.docx]

**Table 10. Philosophical Critiques of CP**

**CP is Insufficient to Capture Emotional States**

"I **don't think that you can detect emotion in that kind of automatic way**... People have spent so much time trying to do it. There are quite good scholars of emotions... who argue persistently and consistently, that that's not how emotions work, that's not how affect [works]. **It is not something that is measurable purely by a physiological impulse**...So **if you think that these technologies are going to... finally solve the problem of subjectivity within mental health research, it's not going to**." (ELPP_01)

"I worry that **we're reducing something that's potentially very complex** and that can be shifting over time." (ELPP_12)

“The h**eterogeneous nature of humans and human behavior [and] mental health is a lot more diverse, disparate, and complex** than measuring, perhaps, diabetes…” (ELPP_13)

**CP Cannot Infer Emotion via Behavior**

"I think that in another concern is that **what a computer can measure is not an internal state; but that's what an emotion is,** and so conflating the two [emotion vs. behavior]. **Your face is moving in this way, so this computer's going to assume what that means is you're feeling this way,** I think it's a concern... **There's going to need to be some interpretation of that data.** Much like a pathologist reads a ultrasound or an MRI or something, **there's still a human interpreting that data**, I think they'll always need to be, because t**hose two things are not the same, an internal state, an outward expression of it**. "(C_19)

"Well, **what worries me right off the bat is how you described what these technologies do, because they don't do any of those things. No technology recognizes, interprets or processes affect.** What these technologies do is they detect movements, of a sort... physical signals that are derived from movements. Maybe they're movements of the face, maybe they're movements of the heart, maybe they are the acoustics of a voice, but **they're not recognizing anything that is related to affect.**" (ELPP_03)

**CP Algorithms Embed Human Biases**

"**You can only train an algorithm on data that is pre-coded by a human being**. Someone has to do that manual human labor first. And digital phenotyping, no matter how much they sort of claim, **they can't get away from that problem**." (ELPP_01)

**CP Inferences not More Valuable than Subjective, Patient Insights**

**"Why do we assume that this kind of data that we might collect is going to be more objective or more reliable than engaging with patients about the phenomenological experiences of their illness?** I think that's a bigger question. It's like, okay, well yeah, maybe the data is helpful, **but it's not necessarily better than what people actually tell us about their experiences. They should have equal footing when we're trying to understand illness and health." (ELPP_16)**

**CP Reflects (Erroneous) Techno-Solutionism**

"I certainly see there's a lot of potential for these technologies, but **my worry is that we have a conversation led by a certain perspective… this idea of solutionism or technological solutionism… So that causes me a lot of concerns, because what we then do is we start to shape these social, political, cultural healthcare questions based around a tech perspective. And what that does is it overlooks a lot of issues that we see." (ELPP_14)**
